# Inefficiency and inequity of the law review submission system

**DOI:** 10.1371/journal.pone.0351410

**Published:** 2026-06-15

**Authors:** Chad M. Topaz

**Affiliations:** 1 Williams College, Williamstown, Massachusetts, United States of America; 2 University of Colorado–Boulder, Boulder, Colorado, United States of America; 3 QSIDE Institute, Williamstown, Massachusetts, United States of America; Ningbo China Institute for Supply Chain Innovation, CHINA

## Abstract

Where a legal scholar works shapes publication outcomes nearly as much as what they write. Law reviews, the primary publication market for legal scholarship in the United States, are run by student editors who face thousands of submissions for a handful of slots and rely heavily on institutional prestige as a proxy for article quality. We build a calibrated agent-based simulation of this market and benchmark it against deferred acceptance, a centralized matching algorithm used in markets like medical residencies. The simulation predicts severe misallocation: more than 60% of top-tier placements differ from what centralized signal-based matching would produce, and the rank correlation between article quality and journal prestige is 0.45 versus 0.79 under centralized matching. Which system produces better placements overall depends on how many authors are competing for how many slots. As competition intensifies, the current system’s disadvantage grows, with the model predicting up to 13.4% loss in match quality. Partial reforms like extending deadlines have negligible effects; in the simulation, the primary source of inefficiency is the decentralized structure of the market itself. The simulation also reveals that credential dependence produces inequity that persists even among articles of comparable quality: authors from prestigious institutions receive markedly better placements regardless of the matching mechanism. Centralized matching fixes the sorting problem but not this equity problem, because prestige bias is embedded in editorial signals and would require changes to how articles are evaluated, not just how they are assigned.

## Introduction

The primary publication venues for legal scholarship in the United States are law reviews: journals edited and managed not by faculty experts but by law students, typically in the second and third years of their legal education [1]. Student editing is, as far as we are aware, unique in the academic world. In most other major disciplines, recognized authorities in the field edit scholarly journals and select manuscripts through peer review [2]. The law review system instead operates as a decentralized matching market, one in which journals and authors make independent offers and acceptances with no central body reviewing all preferences and assigning articles simultaneously. Hundreds of authors simultaneously submit to dozens or hundreds of journals. Student editors evaluate manuscripts under severe time pressure, using author credentials as proxies for quality. Cascading offers propagate up the prestige hierarchy through strategic “trading up,” in which authors accept a better offer and withdraw from a lower-ranked journal [3,4].

The law review system has attracted sustained criticism from within the legal academy [1,4], but no one has quantified the nature and magnitude of its costs. Several features of the market suggest substantial inefficiency. Submission volumes dwarf publication capacity, making it impossible for editors to read every manuscript carefully [5,6]. Surveys show that student editors rely heavily on author credentials as proxies for quality, particularly at higher-ranked journals [5]. Offers frequently carry compressed deadlines, sometimes as short as one hour, and authors routinely use lower offers to obtain expedited review at higher-ranked venues [3,7,8]. The result is a market marked by congestion, noisy signals, and sequential clearing under time pressure. The market design literature identifies such conditions as breeding grounds for coordination failure, where individual actors making separate decisions end up worse off than if they had coordinated. The law review system fits this pattern: journals and authors all act independently, producing mismatches that would not occur under centralized assignment.

Markets sometimes develop problems when participants worry that if they delay, the best options will disappear. In some job markets, for example, employers began making offers earlier and earlier each year, forcing candidates to commit before they had time to evaluate properly. Economists have documented this pattern, called “unraveling,” across many professional markets, from medical residencies to federal clerkships to gastroenterology fellowships [9,10]. Gale and Shapley [11] proved that in any market where two groups must be matched to each other, such as students to schools or articles to journals, there exists an algorithm called *deferred acceptance* that collects everyone’s ranked preferences and makes all assignments simultaneously. The result is what economists call a *stable* matching: an assignment in which no unmatched article–journal pair would both prefer each other over their current arrangement. Put differently, there are no “obvious trades” left that both sides would want to make. Centralized clearinghouses based on stable matching have resolved unraveling in many of these markets [12,13]. Laboratory experiments confirm the pattern: when market rules permit take-it-or-leave-it offers with short deadlines, markets clear inefficiently early, and the resulting matches are of lower quality than those produced under rules that allow open offers or renegotiation [14]. Theoretical work shows that unraveling worsens when participants have poor information and when demand exceeds supply [15]. The law review market exhibits both conditions: editors have limited time to read submissions, and submission volume far exceeds publication capacity. At a general level, successful marketplaces must attract enough participants, reduce congestion so that participants have time to assess each other carefully, and use rules that allow participants to express their honest preferences without fear [13]. The law review system, combining mass simultaneous submission, overwhelmed student editors, and take-it-or-leave-it offers, fails on all three counts.

Legal scholars have extensively documented the institutional structure and history of student-edited law reviews [1,2]. Christensen and Oseid [5] established that editors rely heavily on author credentials rather than carefully reading every article. Dorf [3] characterized the strategic dynamics of expediting and trading up, and reform proposals have focused primarily on extending or standardizing offer deadlines [3,4]. Most recently, Arft-Guatelli and Baker [16] have proposed replacing the current system with a centralized clearinghouse modeled on the medical residency match, drawing on institutional parallels with other two-sided matching markets. What this literature lacks is a formal quantitative framework for evaluating how much inefficiency the current system actually produces, which institutional features are responsible, and how alternative mechanisms would perform. Without such a framework, debates about reform have proceeded on the basis of anecdote and institutional intuition rather than systematic comparison.

We address this gap by developing an agent-based simulation: a computer model that represents individual journals and authors as separate units called “agents,” each following realistic behavioral rules. Their independent decisions interact to produce market-level outcomes. We chose this approach because it lets us represent 200 journals with detailed variation, which simpler mathematical models cannot do. We calibrate the simulation to empirical data on submission volumes, editorial behavior, and institutional practices, incorporating the key features of the current system described above. We compare the current system against the Gale–Shapley deferred acceptance algorithm [11] and several partial reforms, using 2,000 simulation runs with shared random inputs. Each mechanism faces the same hypothetical population of journals and articles, so differences between them reflect the mechanism design itself, not random variation in the simulated population.

Five findings emerge from the simulation. First, centralized matching produces far better sorting of articles to journals: the rank correlation between article quality and journal prestige—a measure of how well the best articles land in the best journals—is 0.79 under centralized matching versus 0.45 under the current system, and more than 60% of top-tier placements differ from what centralized signal-based matching would produce. Second, which system produces better placements overall depends on market competitiveness—when publication slots are scarce relative to submissions, centralized matching dominates, and its advantage grows as competition intensifies, a trend submission data suggest is already underway [17]. Third, the current system compresses quality across the journal hierarchy: elite journals receive substantially weaker articles than they would under centralized matching. Fourth, extending offer deadlines alone has negligible effects; the primary source of misallocation is the decentralized structure of the market itself. Fifth, credential dependence produces inequity that persists even among articles of comparable quality: authors from prestigious institutions receive markedly better placements regardless of the matching mechanism, and centralized matching cannot fix this bias.

## Background

The law review submission system differs from scholarly publishing in most other academic disciplines in two key respects: student editors rather than faculty experts select manuscripts, and authors submit simultaneously to dozens or hundreds of journals rather than one at a time. This section describes the institutional features that our model formalizes.

### From peer review to student editing

In most fields of science, social science, and the humanities, scholarly publishing rests on two principles. First, authors submit to a single journal at a time, a practice called *exclusive submission* [18]. Second, faculty experts evaluate manuscripts through *peer review*, providing detailed reports that advise the editor to accept, revise, or reject [2]. The process commonly takes several months [18]; the editor who makes the final decision is typically a senior scholar in the field.

American law reviews depart from both of these norms. The oldest continuously published student-edited law review, the *Harvard Law Review*, was founded in 1887; by 1930 the model had become standard at American law schools [1,2]. The number of such journals has grown enormously since then: current data from Scholastica, a major electronic submission platform, records 747 law reviews, of which 195 are general (flagship) law reviews and 542 are specialty journals [4,17]. This historical shift from faculty expert review to student editing created the conditions for the market-design problems this paper investigates.

### The submission and selection process

Unlike the exclusive-submission norm of other disciplines, the default practice in the law review market is simultaneous submission: authors submit the same manuscript to dozens or even hundreds of journals at once [4]. Electronic submission platforms—primarily Scholastica, and historically ExpressO—have made this simple and inexpensive [4].

Some journals have introduced exclusive submission tracks as alternatives. Columbia Law Review welcomes ten-day exclusive submissions [8], and the University of Pennsylvania Law Review offers seven-day exclusive windows [7]. Scholastica’s current author guidance describes exclusive-submission tracks as a growing practice [17]. However, simultaneous nonexclusive submission remains the dominant mode, and the system’s characteristic dynamics—expediting, trading up, and cascading offers—arise precisely because of it.

The result is an enormous volume of submissions relative to available publication slots. The Georgetown Law Journal, for instance, receives over 2,000 submissions per year [6]. One Top 25 journal in the Christensen and Oseid [5] study reported receiving 2,219 submissions in a single year. Yet individual journals publish relatively few articles: a 2004 estimate placed the figure at 4–20 unsolicited articles per year [4], and a spot check of recent volumes confirms that flagship journals still publish on the order of 10–20 articles annually (e.g., the *Yale Law Journal*, Volume 134, contains fourteen articles across eight issues; authors’ count from table of contents). Scholastica platform data show that roughly 1 in 15 submissions is accepted [17]. The competitive pressure this creates shapes nearly every aspect of the selection process.

Because student editors lack the deep subject-matter expertise of faculty peer reviewers, and because the volume of submissions is overwhelming, the selection process operates very differently from peer review in the sciences. In many fields, author identity is hidden from reviewers through “blind review” to reduce bias (though practices vary, and single-blind or open review is common in some disciplines). The traditional model of law review selection takes the opposite approach: many journals require authors to submit a resume or curriculum vitae alongside the manuscript, and editors review these credentials alongside the submission [4]. Georgetown and Penn, for example, explicitly request a CV with submissions [6,7]. Current practice is more varied than this traditional model suggests. Harvard Law Review asks authors to enable anonymous review [19]. Washington Law Review states that its review process is anonymous until the final vote, with the separately submitted CV not visible to early-round readers [20]. Columbia Law Review says it strongly prefers subjecting submissions to peer review where time permits [8].

Nonetheless, the empirical evidence shows that author credentials function as a significant proxy for article quality at many journals, particularly higher-ranked ones. Christensen and Oseid [5] surveyed student editors across all tiers of law schools and found that editors are heavily influenced by author credentials. Their data show that 83–100% of editors at Top 25 journals reported being influenced by where an author currently teaches (Table 1 in their study). Editors also consider where an author graduated from law school and the number and placement of the author’s prior publications [5]. Manley [4] puts the dynamic bluntly: students often rely on observable proxies for quality, such as where the author works, rather than reading deeply to assess article merit. The degree of reliance on credentials varies across journals and tiers, but the pattern is consistent across available surveys.

**Table 1:** Model parameters. Each row lists a parameter, its baseline value, the range explored in sensitivity analyses (if any), and its calibration source. Parameters marked “Robustness” are varied to test whether conclusions change; others are held constant. The text explains all key parameters in detail.

Parameter	Symbol	Baseline	Sensitivity	Source
*Market structure*				
Journals	*J*	200	—	Scholastica (2026)
Articles per cycle	*N*	800	—	Informed by Scholastica (2026)
Market tightness	λ	1.0	0.25–1.0	Robustness
Slots (Tier 1/2/3/4)	*s*_*j*_	3–6/4–8/5–10/6–12	—	Manley (2017); Scholastica (2026)
Time steps	*T*	60	—	Exceeds 47-day avg. rejection [17]
*Agent attributes*				
Quality distribution	$q_{i}\sim Beta(\alpha ,\beta )$	(2, 5)	—	Christensen & Oseid (2007)
Prestige noise	$\epsilon_{i}\sim Beta(\alpha^{\prime },\beta^{\prime })$	(2, 5)	—	Modeling choice
Prestige–quality mixing	ρ	0.4	0.1–0.8	Modeling choice; robustness 0.1–0.8
Submission $\ell_{i}$		𝒩(μ=1,σ=5)	—	Modeling choice
Submission width		$Exp(30+(1-p_{i})\cdot 50)$	—	Calibrated to congestion patterns
Aspiration scale	*γ*	0.4	0.2–0.6	Modeling choice
*Editorial evaluation*				
Signal quality weight	$𝔼[w_{j}]$ by tier	0.50/0.55/0.65/0.70	—	Christensen & Oseid (2007)
*w*_*j*_ concentration		25	—	Modeling choice
Signal noise	σ	0.15	—	Modeling choice
Review capacity (T1/2/3–4)	*c*_*j*_	30–50/20–40/10–30	—	Christensen & Oseid (2007)
*Institutional rules*				
Deadlines (Tier 1/other)	*d*_*j*_	{1,3,5,7}/{1,1,2,3}	14 (uniform)	Informed by Dorf (2012); journal policies
Expedite responsiveness	$\pi_{j}\sim Beta$	μ=0.65, κ=8	—	Informed by journal policies
Overbooking (T1/2/3/4)	$\omega_{j}$	1.5/2.0/2.5/3.0	—	Christensen & Oseid (2007)
Deadline behavior		accept	{accept, lapse}	Robustness
*Simulation*				
Monte Carlo replications	*B*	2,000	—	Convergence diagnostics, Fig 2

The time editors spend reading a submission before making a decision reinforces this point. In the Christensen and Oseid [5] survey, a substantial fraction of editors at higher-ranked journals reported spending 30 minutes or less reading an article before reaching a publication decision (see their Fig 3). At the volume of submissions these journals receive, more thorough review of every manuscript is not feasible.

When a journal decides to publish an article, it extends an *offer* to the author, typically with a limited *acceptance period* during which the author must respond. Deadline practices vary sharply. Some journals impose “exploding offers” with deadlines as short as one hour, in which the author must decide immediately with almost no time to shop around [3,7,8]. A coalition led by the *Harvard Law Review* committed to seven-day windows [3], and the University of Chicago Law Review says it will likely grant extensions [21]. The result is wide variation in deadline practices even among elite journals. Unlike other disciplines, where exclusive submission norms mean that an offer of publication is nearly always accepted [18], in law reviews an offer is often merely the opening move in strategic negotiation.

### The expedite system and trading up

The acceptance period creates the conditions for *expediting* and *trading up*, arguably the most distinctive and consequential feature of the law review submission system. An author who receives an offer from a lower-ranked journal does not simply accept it. Instead, the author contacts higher-ranked journals to which the same manuscript was simultaneously submitted and requests an *expedited review*, using the existing offer as evidence that another journal found the work publishable. The higher-ranked journals, which may not have yet read the manuscript, now have a reason to prioritize it: the existence of a competing offer serves as a signal of quality, and the expiring acceptance period creates urgency. If a higher-ranked journal makes a counteroffer, the author accepts it and withdraws from the original journal—a practice known as *trading up* [3,4].

Authors have developed a clear multi-step strategy for trading up. Dorf [3] describes the strategy explicitly: (1) get an offer from a low-ranked journal; (2) use it to request expedited reviews at higher-ranked journals; (3) get an offer from a mid-ranked journal; (4) use that offer to expedite at even higher-ranked venues; and so on up the hierarchy. Christensen and Oseid [5] document significant frustration among editors about trading up, with editors reporting that authors routinely withdraw accepted manuscripts to publish in higher-ranked venues.

Journals vary in how they respond to expedite requests. Many do prioritize expedited articles for faster review, and Scholastica’s guidance to authors suggests that top reviews are often more responsive when the triggering offer comes from a similarly ranked journal [17]. However, some journals explicitly state that expedited review confers no competitive advantage; the University of Pennsylvania Law Review [7] and NYU Journal of International Law and Politics [22], among others, make this claim. Others, such as the University of Chicago Law Review [21] and Washington University Law Review [23], say only that they will attempt to honor expedite requests. How much expediting actually accelerates review therefore varies widely across the market.

The result is a cascading process in which offers propagate upward through the journal hierarchy, with lower-ranked journals frequently losing articles they have already accepted. Manley [4] observes that more prestigious journals, seeing that an offer has been made, assume it must be of reasonably high quality and quickly prioritize it for review. This dynamic may further reinforce credential-based selection if articles by authors at elite institutions are more likely to generate the initial offers that trigger the cascade. These interlocking features—simultaneous submission, credential-based screening, compressed deadlines, and cascading trading up—create substantial potential for misallocation, which we quantify in the model developed next.

## Model

We model the law review submission and placement process as an agent-based simulation in which individual journals and authors follow specified behavioral rules, interact over discrete daily time steps, and produce market-level outcomes. Each simulation run represents a single submission cycle. We chose this approach because it preserves the detailed ranking hierarchy of 200 journals while allowing each journal and author to behave differently—a level of heterogeneity that is central to analyzing how placements differ across the full hierarchy.

In broad outline, the simulation works as follows. At the start of each run, 800 articles are submitted to 200 journals. Each journal reviews articles from its queue, spending more time on promising submissions and less on weak ones. Journals extend offers with deadlines; authors hold offers and use them to request expedited review at higher-ranked journals; when a better offer arrives, authors trade up and withdraw from the previous journal. This process repeats daily for 60 simulated days. We then compare the resulting placements against those produced by centralized matching and several partial reforms. The remainder of this section specifies each component of the model in detail.

### Agent populations

We describe each component of the model below in plain text, with the value and calibration source of each parameter explicitly noted. Table 1 provides the canonical summary of the major model parameters—those governing market structure, agent attributes, editorial evaluation, institutional rules, and the simulation design—together with their baseline values, sensitivity ranges, and calibration sources. A small number of implementation constants (the asymptotic bounds and slope of the quality-dependent effort curve in Eq 3, the standard deviation of author-level aspiration noise, the coefficients of the time-dependent acceptance threshold in Eq 4, the floor on submission width, and the percentile threshold used in the editorial-triage robustness check) are introduced and justified at the point where they appear in the prose below. Parameters explicitly varied to test robustness are marked accordingly in Table 1 and explored in either the *Results* or the *Robustness checks* subsections; parameters held constant at baseline values are justified individually in the surrounding prose.

*Journals*. Each journal j∈{1,2,…,J} is characterized by a prestige rank *r*_*j*_, where $r_{j}=1$ denotes the most prestigious journal and $r_{j}=J$ the least. We set *J* = 200, representing the approximate number of general-interest law reviews that participate meaningfully in the submission and expedite system. We convert rank to a prestige score using a linear formula: $g(r_{j})=1-(r_{j}-1)/(J-1)$. Under this formula, the top-ranked journal ($r_{j}=1$) scores 1, the lowest-ranked journal ($r_{j}=200$) scores 0, and everything in between receives a proportional value.

To manage the complexity of 200 journals, we partition them into four tiers that recur throughout the model: Tier 1 (Elite, ranks 1–20), Tier 2 (Top, ranks 21–50), Tier 3 (Upper-Middle, ranks 51–100), and Tier 4 (Lower, ranks 101–200). These boundaries roughly correspond to the categories used in Christensen and Oseid’s [5] survey of editorial practices (Top 25, Top 50, Top 100, and below). The tier sizes increase down the hierarchy (Elite: 20 journals, Top: 30, Upper-Middle: 50, Lower: 100), giving the model finer resolution at the top of the hierarchy where placement competition is most intense. We draw several journal attributes from tier-dependent distributions at the start of each simulation run.

**Fig 1:**
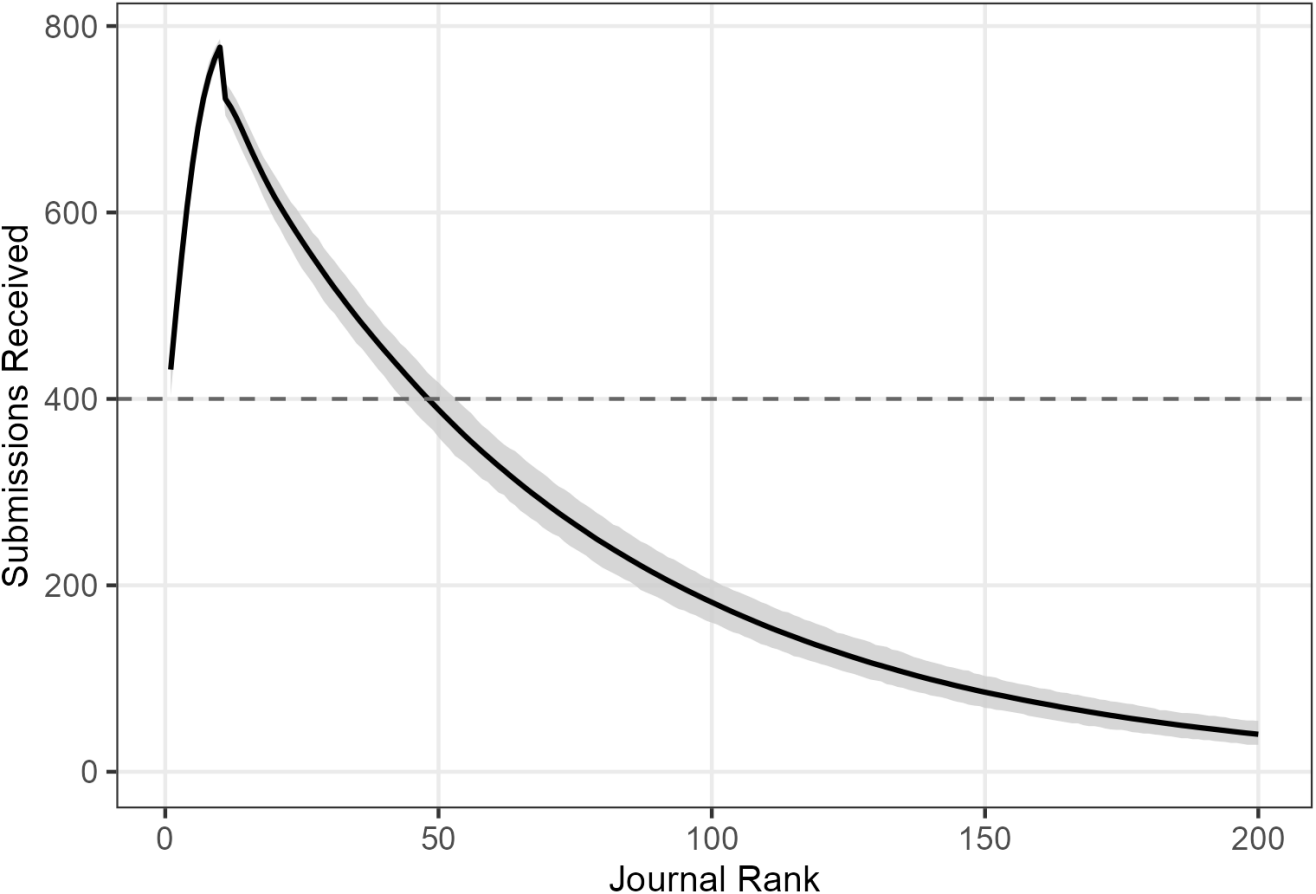
Simulated submissions per journal rank. Solid line shows the mean number of submissions received per season at each journal rank; shaded band shows the 95% range across 500 simulation runs. All counts reflect the model’s scaled population of *N* = 800 articles per season; real-world annual volumes are proportionally larger because the model represents one of two annual submission seasons and uses a subset of the national author population. The dashed horizontal line marks the Georgetown Law Journal’s reported annual volume of approximately 2,000 submissions [6] scaled to the model population for visual reference. The scaling uses a working reference of roughly 4,000 authors in the national annual flagship market, giving a reference line at 2,000×(800/4,000)≈400 submissions at the model scale. We calibrate the model to produce a top-heavy submission shape comparable to documented patterns at rank-25 journals rather than to match Georgetown’s volume exactly. See the Model section for the per-journal congestion targets used.

*Publication slots*. Each journal has a number of publication slots *s*_*j*_—available spots for articles—in a given cycle. Journals publish roughly 10–20 articles per year across two main seasons [4,17]. Because the model represents a single 60-day season, we calibrate per-cycle slots as $s_{j}\sim Uniform(3,6)$ for Tier 1, Uniform(4, 8) for Tier 2, Uniform(5, 10) for Tier 3, and Uniform(6, 12) for Tier 4. Top-ranked journals tend to publish fewer, longer articles, while lower-ranked journals publish more [5]. We vary how competitive the market is using a *market tightness* parameter λ. At the baseline λ=1.0, the market is moderately loose: there are about two slots per article. Lower values of λ represent tighter markets where fewer slots are available relative to submissions, creating more intense competition. The parameter scales all slot counts: the effective slot count for each journal is $\max(1,round(s_{j}\cdot \lambda ))$. At baseline, the 200 journals collectively offer approximately 1,545 slots for 800 articles, nearly two slots per article. Although the aggregate ratio exceeds unity, the top-heavy submission distribution (see Fig 1) ensures that elite journals face intense competition—the Georgetown Law Journal alone receives over 2,000 submissions per year [6] for roughly 8–10 slots [4]—while lower-ranked journals may have unfilled capacity. The market tightness parameter λ allows us to explore tighter regimes where this ratio falls well below unity.

*Review capacity*. Each journal has a review capacity *c*_*j*_ representing the amount of editorial attention available per day. We measure this in abstract “effort units”—a convenience for the simulation. One effort unit represents roughly either a full, careful read of a promising article or a quick screening of about three weak ones. This captures the realistic pattern that editors spend more time on promising submissions and less on obviously weak ones. We draw the ranges from tier-dependent uniform distributions: $c_{j}\sim Uniform(30,50)$ for Tier 1, Uniform(20, 40) for Tier 2, and Uniform(10, 30) for Tiers 3 and 4. Elite journals have larger editorial boards and more organized review committees, giving them greater total editorial capacity per unit time [5]; the tier gradient reflects this pattern. The specific ranges are calibrated so that, in combination with the quality-dependent effort cost, journals review a realistic fraction of their queue each period rather than clearing it instantly or leaving it nearly untouched.

*Offer deadlines*. When a journal extends an offer, it specifies a deadline *d*_*j*_ (in time steps) by which the author must respond. As documented above, deadline practices vary sharply by journal and by offer context, with both elite and lower-ranked journals sometimes using very short deadlines. For Tier 1 journals, *d*_*j*_ is drawn uniformly from {1, 3, 5, 7}, spanning the range from same-day exploding offers to the seven-day window announced by the *Harvard Law Review* coalition [3]. For all other tiers, *d*_*j*_ is drawn from {1, 1, 2, 3} (with duplication reflecting the higher frequency of very short deadlines). The key variation is within-tier, not simply between tiers.

Because the model uses one-day time steps, all sub-day deadlines—including the one-hour expedited-offer deadlines documented at Penn and Columbia—are collapsed to a single time step ($d_{j}=1$). This tractability simplification may understate the pressure in the actual market. In reality, a one-hour “exploding offer” and a twenty-four-hour deadline impose very different strategic constraints, since with one hour an author has almost no time to seek better offers. A finer time resolution would better capture this distinction but would substantially increase computational cost with unclear gains for the mechanism comparisons that are the paper’s primary focus.

*Overbooking factor*. Just as airlines sell more tickets than seats, journals may extend more offers than they have slots, anticipating that some authors will trade up and withdraw. The overbooking factor $\omega_{j}$ caps the total number of committed positions—both pending offers and accepted articles—at $\lceil s_{j}\cdot \omega_{j}\rceil$ (the product of slots and the overbooking factor, rounded up). We set $\omega_{j}=1.5$ for Tier 1, 2.0 for Tier 2, 2.5 for Tier 3, and 3.0 for Tier 4. The tier gradient reflects the empirical finding that lower-ranked journals lose a larger fraction of accepted articles to trading up and must therefore overbook more aggressively [5]. The specific multipliers are heuristic: Tier 1 journals, which sit at the top of the hierarchy and lose relatively few authors to upward moves, need only modest overbooking (50% above capacity), while Tier 4 journals, which face the highest withdrawal rates, may extend up to three times their slot count. The exact values are not individually calibrated to data; what matters for the simulation is the qualitative pattern—increasing overbooking down the hierarchy—and the resulting aggregate dynamics, which are robust across reasonable alternative specifications.

*Signal weight (journal heterogeneity)*. Different journals have different editorial standards. Some elite journals rely heavily on author credentials when evaluating articles; others try to focus more on article content. We capture this variation with a weight $w_{j}\in (0,1)$ for each journal that governs the balance between article quality and author prestige in its editorial evaluation (see the signal structure below). A weight of 1 means the journal evaluates purely on article quality; a weight of 0 means purely on author prestige; intermediate values blend the two. We draw each *w*_*j*_ independently from a Beta distribution centered on a tier-specific mean (standard deviation ≈0.08), so journals within the same tier differ but cluster near their tier average. The tier means are $𝔼[w_{j}]=0.50$ for Tier 1, 0.55 for Tier 2, 0.65 for Tier 3, and 0.70 for Tier 4. Lower values mean greater reliance on author prestige. This captures the empirical pattern documented by Christensen and Oseid [5]: editors at the highest-ranked journals rely most heavily on author credentials, while editors at lower-ranked journals place relatively more weight on article content. We note that *w*_*j*_ is a rough simplification, not a structural representation of each journal’s review process. Journals that use stage-structured anonymous review, such as Harvard, Chicago, and Washington, reveal author identity only in later rounds, which *w*_*j*_ does not capture. The tier-dependent means directionally reflect variation in how much editorial assessment depends on author identity, but the mapping from institutional review structure to a scalar weight is necessarily approximate.

*Articles and authors*. Each author i∈{1,2,…,N} submits one article per cycle. We set *N* = 800. Scholastica platform data confirm that law review submissions concentrate into two main annual seasons—spring (beginning around February 1) and fall (beginning around August 1)—with over half of all submissions occurring in the six weeks following these dates [17]. The actual annual volume of submissions across all U.S. law reviews has been estimated at several thousand authors [4], with individual elite journals receiving over 2,000 submissions per year [6]; *N* = 800 therefore represents one season of submissions for a model population that is proportionally smaller than the full national market.

We calibrate the simulation *proportionally* to per-journal congestion patterns rather than to absolute submission volume. Most model parameters are anchored to per-journal or per-author quantities that do not depend on *N*. Per-cycle slot counts *s*_*j*_ are calibrated to the empirical 10–20 unsolicited articles per year [4], per-day review capacity *c*_*j*_ to editorial-board size and effort patterns documented by Christensen and Oseid [5], and the signal noise σ, the quality and prestige distributions, the aspiration scale γ, and the time horizon *T* = 60 to similarly *N*-independent quantities. The only parameters implicitly tied to *N* are the author submission-width parameters, which we calibrate to produce a top-heavy submission distribution: roughly 71% of simulated authors include rank-25 journals in their submission set, generating per-journal queues at the high-end of the hierarchy that are dense enough to reproduce the congestion regime documented by Christensen and Oseid [5] for Top 25 journals (Fig 1). This calibration targets the qualitative congestion pattern rather than exact volume; absolute submission counts in the model are smaller than in the real annual market both because *N* = 800 represents one of two annual submission seasons and because the model uses a subset of the national author population.

Under this calibration, the comparison between mechanisms (current system versus deferred acceptance versus partial reforms) depends on the slot-to-article ratio and the structural dynamics of cascading offers, not on the absolute scale. The model’s market-tightness sensitivity (see the Market Tightness subsection in Results) varies the slot-to-article ratio across a four-fold range (λ∈[0.25,1.0]). Because halving the per-journal slot count is equivalent in competitive pressure to doubling *N* while holding slots fixed, our λ∈[0.25,1.0] sweep tests effective competition levels equivalent to N∈[800,3,200], a broad range of effective competition that reaches volumes approaching the several-thousand-author national annual scale. The substantive conclusions—that decentralized clearing sorts substantially worse than centralized matching, and that the gap grows as competition tightens—are robust across this range.

Computational tractability also motivates the choice of *N* = 800. The per-replication cost of the agent-based simulation scales with N·J·T, and the baseline Monte Carlo (*B* = 2,000 replications), the four sensitivity analyses, and the 399-cell heatmap grid (each cell at 200 replications) together make computational efficiency a binding consideration. We chose *N* = 800 as a population large enough to produce stable per-journal dynamics that match the empirical proportional calibration, while keeping the full Monte Carlo pipeline tractable across all sensitivity analyses. One quantity that scaling *N* upward *would* change is the absolute volume of each journal’s queue, which in reality would push editors to triage more aggressively; the robustness check on explicit desk-rejection (see Robustness checks) directly addresses this regime.

Each article is characterized by the following attributes.

*True quality*. Each article has a true quality *q*_*i*_ on a 0–1 scale, drawn from a distribution skewed toward lower quality: most articles are of modest quality, and only a small fraction are excellent. This matches what editors report in surveys [5]. We use the Beta(2, 5) distribution to produce this skewed pattern.

*Author prestige*. Prestige $p_{i}\in [0,1]$ captures the institutional standing of the author, including the reputation of their university and their publication record. Prestige and quality are related but different: a famous professor may write a mediocre article, and an unknown scholar may write an excellent one. We model prestige as a blend: part of an author’s prestige comes from their actual article quality, and part comes from their institutional reputation and career stage—factors unrelated to this particular article. The weight ρ controls how much prestige tracks quality; it is a blending weight, not a correlation coefficient. Formally,


$$\begin{array}{c} p_{i}=\rho \cdot q_{i}+(1-\rho )\cdot \epsilon_{i},\;\epsilon_{i}\sim Beta(2,5), \end{array}$$ (1)

constrained to fall in the range [0,1]. We draw the random component $\epsilon_{i}$ from the same skewed Beta(2, 5) distribution as article quality so that both variables share the same skewed shape (most values modest, a few high). At the baseline ρ=0.4, the resulting correlation between prestige and quality is approximately 0.56, meaning prestige is a noisy but nonzero signal of article quality. Because ρ governs how much the model’s results depend on credential-based screening, it is varied across {0.1,0.2,…,0.8} in robustness checks.

*Submission set*. Each author submits to a range of journals ranked from their most-preferred target (the best journal they hope to get into) to a least-preferred fallback. The range is contiguous—a ranked sequence with no gaps—because if an author is willing to submit to journal X and to a lower-ranked journal Y, they would also submit to everything in between; skipping makes little sense when the marginal cost of one additional submission is only a few dollars [17]. Formally, each author submits from a most-preferred target $\ell_{i}$ (the highest-ranked journal in their set) down to a least-preferred target *h*_*i*_ (the lowest-ranked). Because the per-journal cost is low, almost every author includes elite journals in their submission set; the main variation is how far *down* the hierarchy an author also submits [4,17]. We model this asymmetry directly.

Almost all authors include elite journals in their submission set. We model this by drawing the top target $\ell_{i}$ from a normal distribution centered at rank 1 with standard deviation 5, rounded to the nearest integer and clamped to [1, *J*]. This does not depend on author prestige: both well-known and unknown authors include elite journals. In the resulting distribution, approximately 55% of authors target the single most prestigious journal, and over 95% target a journal in the top 10.

How far down the hierarchy each author submits—the *width* of their submission set—follows an exponential distribution. This is a probability distribution in which most values cluster at moderate levels but a long tail of larger values is possible. The mean width depends on prestige, capturing the pattern that lower-prestige authors cast a wider net [4]. Formally, ${\text{width}}_{i}\sim \max(10,\;round(X_{i}))$, where $X_{i}\sim Exponential(\text{mean}=\mu_{i})$ and $\mu_{i}=30+(1-p_{i})\cdot 50$. We chose the parameters of this formula to reproduce two empirical targets. First, the top-heavy submission pattern: elite journals receive the vast majority of submissions, with counts declining steadily down the hierarchy. Second, rank-25 journals in the model face dense queues comparable to the Top-25 congestion documented in the Christensen and Oseid [5] survey when the model is scaled to *N* = 800 (Fig 1). High-prestige authors ($p_{i}\approx 0.8$) submit to about 40 journals on average (targeted, elite-focused); authors at the mean prestige ($p_{i}\approx 0.29$) submit to about 65; and low-prestige authors ($p_{i}\approx 0.1$) submit to about 75. Because the exponential distribution is skewed, most authors submit to somewhat fewer journals than these averages, while a tail of authors submit very broadly. The bottom of the submission range is $h_{i}=\min(J,\ell_{i}+{\text{width}}_{i}-1)$.

With these baseline parameters (averaged over 500 replications), the submission count peaks around rank 10 at approximately 780 submissions per season, then declines steadily: rank-25 journals receive approximately 570 per season, rank-50 approximately 390, rank-100 approximately 180, and rank-200 approximately 40. Rank-1 journals receive approximately 430 per season—slightly below the peak because a fraction of authors’ lower bounds $\ell_{i}$ fall above 1. The real-world figure for Georgetown (rank ≈25) exceeds 2,000 submissions per year [6]; in the model, rank-25 receives approximately 1,140 per year. The model’s absolute volume is therefore smaller than the real annual market, reflecting both that *N* = 800 represents one of two annual submission seasons and that the model uses a subset of the national author population. What the calibration does match is the top-heavy proportional shape: approximately 71% of simulated authors include rank-25 journals in their submission set, generating per-journal queues at the top of the hierarchy that are dense enough to reproduce the editorial-time pressure documented by Christensen and Oseid [5]. Fig 1 plots the full submission density curve (mean and 95% band across replications), confirming the monotonic decline from the peak at rank 10 through rank 200.

### Signal structure

Journal editors do not know the true quality of an article. They form an imperfect assessment based on what they can infer from reading it and what they know about the author’s reputation, and they make mistakes. We model this as a noisy blend of article quality and author prestige. Formally, each journal *j* forms an assessment of each article *i* in its submission queue:


$$\begin{array}{c} {\hat{q}}_{ji}=w_{j}\cdot q_{i}+(1-w_{j})\cdot p_{i}+\eta_{ji},\;\eta_{ji}\sim 𝒩(0,\sigma^{2}), \end{array}$$ (2)

where *w*_*j*_ is the journal-specific quality weight described above and σ=0.15 is the standard deviation of the evaluation noise. Because article quality and prestige both lie in [0,1], a noise standard deviation of 0.15 means that a journal’s assessment of a given article is informative but far from perfect: roughly one in four evaluations shifts an article’s perceived quality by more than one-sixth of the full 0–1 range, consistent with the high error rates one would expect from 5–30 minute reads of complex manuscripts. We compute the full set of assessments once at the start of each simulation run. All mechanisms within a run share the same editorial assessments, so when we compare the current system to centralized matching, any differences reflect the design of the placement process rather than random variation in editorial judgment.

The signal structure captures three empirical features documented by Christensen and Oseid [5]. First, journals observe a blend of article quality and author prestige rather than either alone. Second, editors typically spend only 5–30 minutes reading a submission before reaching a decision, so their assessments contain substantial error. Third, journals differ in how much they rely on credentials versus content, with elite journals weighting credentials more heavily.

### Sequence of play: The current system

The simulation proceeds over *T* = 60 discrete time steps, representing a 60-day submission season, which exceeds the 47-day average time journals take to reject submissions [17], ensuring there is enough time for the full market process to unfold. On day 1, all 800 authors simultaneously submit their articles to their respective submission sets. Each journal receives a queue of all articles whose submission range includes that journal. Because all articles arrive at once, the initial ordering within each queue is random. On each subsequent day, the simulation first checks for offers whose deadlines have expired. When an offer deadline expires without an explicit author decision, two things could happen. Under “accept” behavior (the baseline assumption), the author automatically keeps the best offer they are holding. Under “lapse” behavior (a robustness variant), the offer simply withdraws, freeing the journal’s slot and returning the author to the submission pool. We test both assumptions to ensure our conclusions do not depend on this modeling choice.

*Journal review and offers.* We process journals in a uniformly random order at each time step to avoid positional bias. Each journal that has not yet reached its overbooking cap proceeds through three steps: queue prioritization, review, and offers.

*Queue prioritization.* When articles currently holding offers from other journals appear in a journal’s queue, that journal may move them to the front for expedited review. However, not all journals honor expedite requests equally. As described in the Background section, some journals routinely prioritize expedited articles, while others state that expedites confer no competitive advantage. We model this variation by assigning each journal an independent *expedite responsiveness probability* $\pi_{j}$—a number between 0 and 1 that determines how often the journal actually honors expedite requests. If $\pi_{j}=0.65$, the journal speeds up review of expedited articles about two-thirds of the time and ignores the request the other third. Each $\pi_{j}$ is drawn from a Beta(5.2, 2.8) distribution, which has mean 0.65 and standard deviation ≈0.16. We calibrate this parameter to journal submission guidelines: among the journals we surveyed, the majority describe processes consistent with prioritizing expedited articles (e.g., Georgetown, Columbia), while a meaningful minority explicitly disclaim any advantage from expediting (e.g., Penn, NYU JILP). This distribution produces moderate heterogeneity, allowing some journals to almost always honor expedites and others to rarely do so. We do not impose a systematic tier gradient: the available evidence does not clearly support one direction. Elite journals like Penn state that expedited review confers no competitive advantage, while some lower-ranked journals may be more eager to reprioritize in order to attract strong authors. The journal-by-journal draw captures the substantial heterogeneity documented in practice without forcing an unsupported tier ordering.

*Quality-dependent review.* The journal reviews articles from the front of its queue, consuming effort units from its capacity *c*_*j*_. Not all articles consume the same effort. Editors reject articles with weak signals (below-median assessments) quickly, spending roughly 0.3 effort units each. Articles with strong signals (above-median assessments) require careful full review, costing roughly 1.0 effort units. This captures the empirical finding that editors spend only minutes on weak submissions but considerably longer on promising ones [5]. We use a logistic function to model this gradual S-shaped transition from quick rejection to careful review. The exact shape is not crucial; what matters is the pattern: roughly 0.3 effort units for weak articles, 1.0 for strong ones, and gradual variation in between. Formally, if the signals of articles in the queue have median *m* and range *R*, the effort cost for article *i* is


$$\begin{array}{c} e_{i}=0.3+0.7[1+e^{-({\hat{q}}_{ji}-m)/(R/4)}{]}^{-1}. \end{array}$$ (3)

The floor of 0.3 and ceiling of 1.0 are asymptotic bounds; articles at the queue median cost 0.65 effort units, with a smooth logistic transition between the extremes. The slope parameter *R*/4 ensures the transition is gradual. The exact values are not individually calibrated, but the qualitative behavior—cheap screening of weak articles, expensive review of promising ones—is what drives the model dynamics. The journal reviews articles sequentially from its queue until its daily editorial effort capacity *c*_*j*_ is exhausted. Each reviewed article leaves that journal’s queue, so no journal reviews the same article twice, but the article remains in the queues of other journals to which it was submitted.

*Offers.* From all reviewed articles, the journal extends publication offers to those with the highest quality assessments, up to its overbooking cap of $\lceil s_{j}\cdot \omega_{j}\rceil$ minus the number of slots already committed. Each offer carries the journal’s deadline *d*_*j*_.

*Author decisions.* An author who receives an offer from journal *j* compares it to any existing held offer.

If the new offer is from a more prestigious journal than the author’s current best offer (or if the author holds no offer), the author takes the new offer, drops their previous offer (freeing that journal’s slot for another article), and holds the new offer. This is the *trading-up* mechanism: authors can abandon a commitment to a lower-ranked journal when a better offer materializes [3,4].

The author then decides whether to *formally accept* the new offer and exit the market, or to *hold* it and continue seeking better options through expedited review. This decision depends on two things: how good the offer is relative to the author’s aspirations, and how much time remains in the submission season.

Authors do not target all journals in their submission set equally. They have a realistic aspiration—a journal rank they genuinely hope to achieve, based on their prestige and how selective they are willing to be. This aspiration is distinct from the top of their submission range: an author might submit all the way to rank 1 (casting a wide net) but realistically hope to place around rank 50. Formally, each author has an *aspiration rank* *a*_*i*_, computed as ${\tilde{a}}_{i}=round(1+(1-p_{i})\cdot J\cdot \gamma +\nu_{i})$, where γ=0.4 is the aspiration scale parameter. We add author-level randomness via $\nu_{i}\sim 𝒩(0,\text{sd}=10)$. On the 200-journal scale, a standard deviation of 10 ranks means that roughly two-thirds of authors aspire within ±10 positions of what their prestige would predict, introducing realistic variation without overwhelming the systematic prestige gradient. This value is then constrained to fall within the author’s submission range: $a_{i}=\min(h_{i},\max(\ell_{i},{\tilde{a}}_{i}))$. Before clamping, the latent aspirations ${\tilde{a}}_{i}$ are roughly rank 17 for high-prestige authors ($p_{i}\approx 0.8$), rank 58 for average-prestige authors ($p_{i}\approx 0.29$), and rank 73 for low-prestige authors ($p_{i}\approx 0.1$). Because clamping to $\left[\ell_{i},h_{i}\right]$ only binds when the latent aspiration falls outside the submission range, the post-clamping values are similar for most authors.

Authors become less selective as the submission season progresses. Early on, they accept only offers very close to their realistic aspiration, holding lesser offers as leverage for trading up. As the season winds down and the risk of going unplaced grows, they settle for offers further below their ideal. We model this with an acceptance threshold that increases linearly over time:


$$\begin{array}{c} \alpha (t)=0.10+0.50\cdot \frac{t}{T}, \end{array}$$ (4)

which rises from 0.10 at the start of the season to 0.60 at the end. The author accepts an offer from journal *j* if the journal’s rank falls within the top α(t) fraction of the range between the author’s aspiration rank and the bottom of their submission set. Formally, the author accepts if $r_{j}\leq \lfloor a_{i}+\alpha (t)\cdot (h_{i}-a_{i})\rfloor$. To illustrate: an author aspiring to rank 50 with a submission range extending to rank 150 would, early in the season (α=0.10), accept only an offer from rank 60 or better, holding anything worse as leverage for expedited review. By mid-season (α=0.35), the same author would accept rank 85. By the season’s end (α=0.60), the author would settle for rank 110. Even at the end of the season, however, authors never become completely indiscriminate. In the model, unplaced articles primarily result from never receiving an offer rather than from refusing one, since held offers are accepted at deadline expiry under the baseline rule. The acceptance threshold governs the *timing* of formal acceptance—and thus how long authors stay in the market seeking better offers—rather than whether articles are ultimately placed. This captures the strategic cascade described by Dorf [3]: authors hold early offers from lower-ranked journals as leverage and gradually lower their standards as the submission window closes. The aspiration scale γ is varied from 0.2 (more optimistic authors) to 0.6 (more pessimistic) in the aspiration breadth analysis (see Results).

If the new offer is from a *less* prestigious journal than the author’s current best, the author ignores it. After all *T* time steps, the model assigns any author still holding an offer to the journal of their best held offer.

*End-of-season resolution.* Because journals may extend more offers than they have slots, some journals may hold more articles than they can actually publish at the end of the simulation. We resolve this in two phases. First, each overbooked journal retains the articles with the highest signals and releases the rest, freeing those articles for possible placement elsewhere. Second, we give the released articles a second chance: journals are processed in prestige order (most prestigious first), and each journal with remaining open capacity considers the released articles that were originally submitted to it. These candidates are ranked by signal and the top candidates fill the journal’s remaining slots. The reallocation excludes articles that received no offer during the main simulation. This two-phase procedure ensures that every journal ends up with no more articles than it can publish, while giving displaced articles a second chance at placement. The procedure is conservative in the sense that it benefits the decentralized mechanisms by rescuing articles that would otherwise go unplaced; without it, the current system would match fewer articles and the relative advantage of deferred acceptance (DA) would be larger.

### Counterfactual mechanisms

We compare the current system against seven counterfactual mechanisms, each designed to isolate or remove a specific institutional feature.

*Deferred acceptance.* The Gale–Shapley DA algorithm [11] is a centralized procedure that produces a stable matching, one in which no unmatched journal–article pair would both prefer each other over their current assignments. The algorithm works in rounds: (1) every journal with unfilled slots (*s*_*j*_ slots each) proposes to enough top-ranked articles from its preference list to potentially fill its open slots; (2) each article tentatively holds the best proposal it has received and rejects the others; (3) rejected journals move down their preference lists; (4) this repeats until no journal has a new article to propose. Each article ends up with the best journal that proposed to it, and no unmatched journal–article pair would both prefer each other over their current assignments. Because journals propose, the resulting matching is *journal-optimal*: they get the best outcome possible among all stable matchings [9,11]. The DA mechanism uses the same imperfect editorial assessments as the decentralized mechanisms, so any differences reflect the structure of the placement process, not an informational advantage.

The DA benchmark, however, combines two distinct features that the current system lacks: simultaneous clearing across all articles and journals, and perfect ex-ante prioritization of each journal’s submission queue. Our comparison therefore reflects the *joint* impact of these two features and should be interpreted as an upper bound on what centralization alone could achieve without parallel reform of editorial triage processes. The editorial-triage robustness check reported in the Robustness checks section partially probes the prioritization channel: it gives the decentralized current system an explicit desk-rejection capability that removes the worst-signal articles from each journal’s queue (a realistic form of editorial prioritization, though weaker than DA’s full ex-ante ranking of survivors by signal). Even with this realistic triage in place, the gap to DA persists essentially unchanged, indicating that ordinary editorial triage is not sufficient to close the DA gap. A complete test of DA’s perfect ex-ante prioritization would require a “decentralized full-information” variant in which each journal also ranks its surviving queue by signal; we discuss this in the Limitations section as a direction for future work.

We also run an *author-proposing* variant in which authors propose and journals hold: each unmatched author proposes to the most prestigious journal that has not yet rejected them, and journals tentatively hold the best *s*_*j*_ applicants by editorial assessment. This produces the *author-optimal* stable matching—the version most favorable to authors [9,11]. If the journal-proposing and author-proposing versions produce nearly identical results, it means there is essentially only one stable matching in this market, so the choice of who proposes does not matter.

*Partial reforms.* To understand which features drive inefficiency, we test four partial reforms that change the current system one feature at a time. *Extended deadlines* replaces each journal’s varied deadline with a uniform 14-day window, isolating the effect of compressed deadlines on matching quality. *No expedite* leaves deadlines unchanged but removes queue-jumping: journals review articles in the order received, with no prioritization for articles holding offers elsewhere. Authors can still hold offers and trade up; the only difference is that holding an offer does not advance the article in other journals’ queues. *No expedite + extended deadlines* combines the two preceding reforms, serving as the intermediate step in the efficiency loss decomposition and ensuring that each component changes exactly one institutional feature. *No trading up* requires authors to accept the first offer they receive unconditionally—no holding, no expediting, and no trading up. This more aggressive restriction isolates the effect of strategic delay and upward mobility in author decision-making.

*Random assignment.* Articles are randomly assigned to journal slots, subject only to the constraint that each journal fills its *s*_*j*_ slots (or as many as possible if $N<\sum_{j}s_{j}$). This provides a lower bound on match quality, representing a system with no information whatsoever about article quality or journal prestige.

### Welfare measures

The primary measure is *overall match quality*, also called *all-N welfare*:


$$\begin{array}{c} W(\mu )=\frac{1}{N}\sum_{(i,j)\in \mu }q_{i}\cdot g(r_{j}). \end{array}$$ (5)

For each article placed in a journal, we multiply the article’s quality *q*_*i*_ by the journal’s prestige score *g*(*r*_*j*_) (the linear rank-to-score mapping defined in the Model section). Because the payoff is a product, it rewards *assortative matching*: placing a high-quality article in a prestigious journal contributes more than placing the same article in a low-ranked journal and a mediocre article in the prestigious one [11,12]. We divide by the total number of articles *N*, not only the matched ones, so that mechanisms leaving more articles unmatched are penalized. Because the model represents only the general/flagship market of *J* = 200 journals, “unmatched” means unplaced *within this segment*, not unpublished altogether. Some of these articles might find homes in the 542 specialty journals we exclude from the model [17]. We also report a “matched-only” variant, $\bar{Q}(\mu )=(1/|\mu |)\sum_{(i,j)\in \mu }q_{i}\cdot g(r_{j})$, which averages only among placed articles and thus isolates sorting quality from placement volume. We report the number of matched and unmatched articles for each mechanism.

As a secondary measure of sorting quality, we compute the Spearman rank correlation between article quality and journal prestige among matched pairs, which ranges from 0 (no relationship) to 1 (perfect alignment). We also examine distributional measures: mean article quality within each tier, and a *displacement rate*—the fraction of articles placed in a given tier under one mechanism that would end up in a different tier under centralized matching. A high displacement rate means the mechanism is producing a fundamentally different allocation, not just a noisier version of the same one. Finally, we divide authors into four groups by prestige and compute the average journal rank of placement for each group, revealing whether the current system systematically advantages or disadvantages authors at different prestige levels.

*Decomposition method.* To understand which features of the current system drive the welfare gap relative to centralized matching, we decompose the total gap into three components by imagining a sequence of reforms, each removing one feature:


$$\begin{array}{c} \Delta_{\text{expedite}}=W(\text{No Expedite})-W(\text{Current}), \end{array}$$ (6)

$$\begin{array}{c} \Delta_{\text{deadline}}=W(\text{No Expedite + Extended})-W(\text{No Expedite}), \end{array}$$ (7)

$$\begin{array}{c} \Delta_{\text{central}}=W(\text{DA})-W(\text{No Expedite + Extended}), \end{array}$$ (8)

where W(·) denotes the all-*N* match quality (Eq 5) and the DA benchmark is journal-proposing. The first step removes expedite queue-jumping while preserving original deadlines. The second extends all deadlines to a uniform 14 days, isolating the effect of deadline pressure. The third replaces the remaining decentralized process with centralized matching. The sign of the total gap can be positive or negative depending on parameter settings; at baseline, the current system slightly outperforms DA on all-*N* welfare.

By construction, the three components sum to the total gap *W*(DA) − *W*(Current). We present this as an accounting exercise, not a definitive causal decomposition: performing the same reforms in a different order would attribute different shares to each step. We report both journal-proposing and author-proposing DA results separately so that readers can assess how sensitive the benchmark is to which side proposes.

### Simulation design and parameters

Within each simulation run, all mechanisms operate on the same population of journals and articles, the same editorial assessments, the same initial queue orders, and the same random draws (*common random numbers*), so that any difference in outcomes reflects the mechanism design rather than sampling variation. The DA and random mechanisms are partial exceptions: DA uses the same editorial assessments but not sequential queues, and the random mechanism uses no assessments at all.

**Fig 2:**
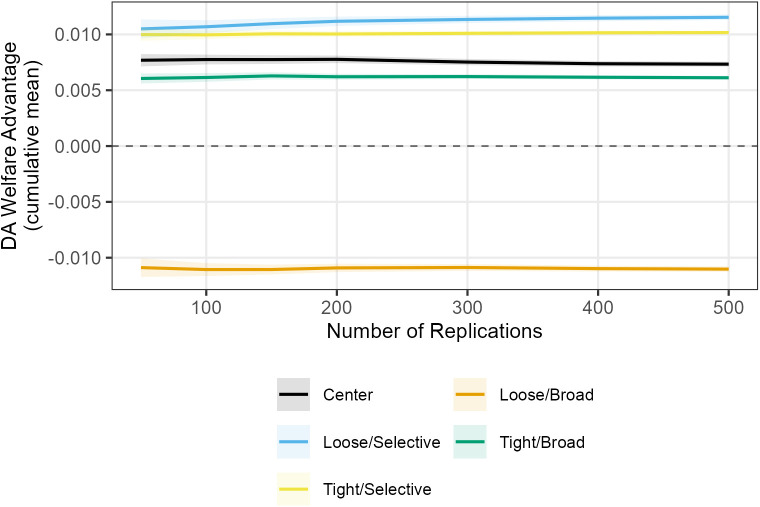
Convergence diagnostics. Cumulative mean of the deferred acceptance (DA) welfare advantage over the current system, plotted against the number of simulation runs completed, for five representative parameter settings. Shaded bands show 95% confidence intervals. All estimates stabilize well before 500 runs.

The simulation is run for *B* = 2,000 independent runs. In each run, a fresh population of journals and articles is generated, editorial assessments are computed, and all eight mechanisms operate on the same population. We then average the results across all 2,000 runs. The large number of runs, combined with the common random numbers design, ensures that our estimates of the differences between mechanisms are precise. To verify that 2,000 runs is sufficient, we check convergence: Fig 2 plots the cumulative mean of the DA welfare advantage as a function of the number of runs completed, for five representative parameter settings from the heatmap grid. All estimates stabilize well before 500 runs, and the 95% confidence bands are narrow, confirming that the 200-replication grid cells and 2,000-replication baseline provide adequate precision. Simulations were implemented in R (version 4.5.1; R Foundation for Statistical Computing, Vienna, Austria) and parallelized across processor cores. Code is available at https://github.com/chadtopaz/law-review.

Table 1 summarizes the major model parameters, their baseline values, the ranges explored in sensitivity and robustness analyses, and their calibration sources; the small number of finer implementation constants is justified in the surrounding prose where each appears.

The parameter most important for the distributional analysis is ρ, the prestige–quality mixing weight, because it determines whether editors’ reliance on credentials is actually reasonable. When ρ is high, prestige is a reliable proxy for quality—credentials are informative, and using them may be relatively harmless. When ρ is low, prestige adds noise to editorial judgments—credentials are misleading, and relying on them introduces substantial mismatches. The sensitivity analysis over ρ directly addresses how the distributional consequences of the current system depend on this relationship.

The market tightness parameter λ scales all journal slot ranges. At λ=1.0 (baseline), the market is moderately loose: total capacity exceeds the number of articles by a factor of approximately 1.9. As λ decreases toward 0.25, the market becomes very tight (approximately 386 slots for 800 articles), and competition for slots intensifies, potentially amplifying the differences between mechanisms. The deadline behavior parameter (accept versus lapse) tests robustness to the assumption about what happens when an offer deadline expires without an explicit author response.

## Results

We organize the results around four questions: how do the mechanisms compare on sorting and throughput (Mechanism comparison), how do placements differ across the journal hierarchy and across author prestige levels (Distributional effects), how sensitive are the findings to market competitiveness and author behavior (Market tightness and Aspiration breadth), and where does the sorting–throughput frontier lie (The sorting–throughput frontier)? The Robustness checks subsection reports robustness checks.

### Mechanism comparison

Table 2 presents the main outcome measures across all eight mechanisms at baseline parameter values. The clearest finding is that centralized matching (DA) produces dramatically better sorting: the rank correlation between article quality and journal prestige rises from 0.449 under the current system to 0.786 under DA—a 75% increase, from a moderate to a strong relationship. Among placed articles, DA also produces higher average match quality (0.272 versus 0.249). These differences are precise: the run-to-run standard deviation is approximately 0.006 for both mechanisms, and the common-random-numbers design ensures that the standard error of the difference is an order of magnitude smaller than the gap itself.

**Table 2:** Baseline aggregate results across mechanisms. “Match Qual.” is the mean of $q_{i}\cdot g(r_{j})$ among placed articles (matched-only welfare); “Rank Corr.” is the Spearman correlation between article quality and journal prestige; “All-*N* Welfare” divides total match quality by all *N* = 800 articles, penalizing mechanisms that leave articles unplaced; “Eff. Gap” is the percentage difference in all-*N* welfare relative to journal-proposing deferred acceptance (DA)—positive values mean the mechanism outperforms DA, negative values mean it underperforms. Values are means across *B* = 2,000 simulation runs; Monte Carlo standard errors are below 0.001 for all welfare entries and below 0.01 for rank correlations.

Mechanism	Match Qual.	Rank Corr.	Matched	All-*N* Welfare	Eff. Gap
Current System	0.249	0.449	488	0.1519	+2.0%
Deferred Accept. (J-prop.)	0.272	0.786	437	0.1489	0.0%
Deferred Accept. (A-prop.)	0.272	0.786	437	0.1489	0.0%
Extended Deadlines	0.249	0.449	488	0.1519	+2.0%
No Expedite	0.249	0.434	490	0.1526	+2.5%
No Expedite + Extended	0.249	0.434	490	0.1526	+2.5%
No Trading Up	0.232	0.294	543	0.1565	+5.2%
Random	0.128	−0.001	800	0.1280	–14.0%

**Fig 3:**
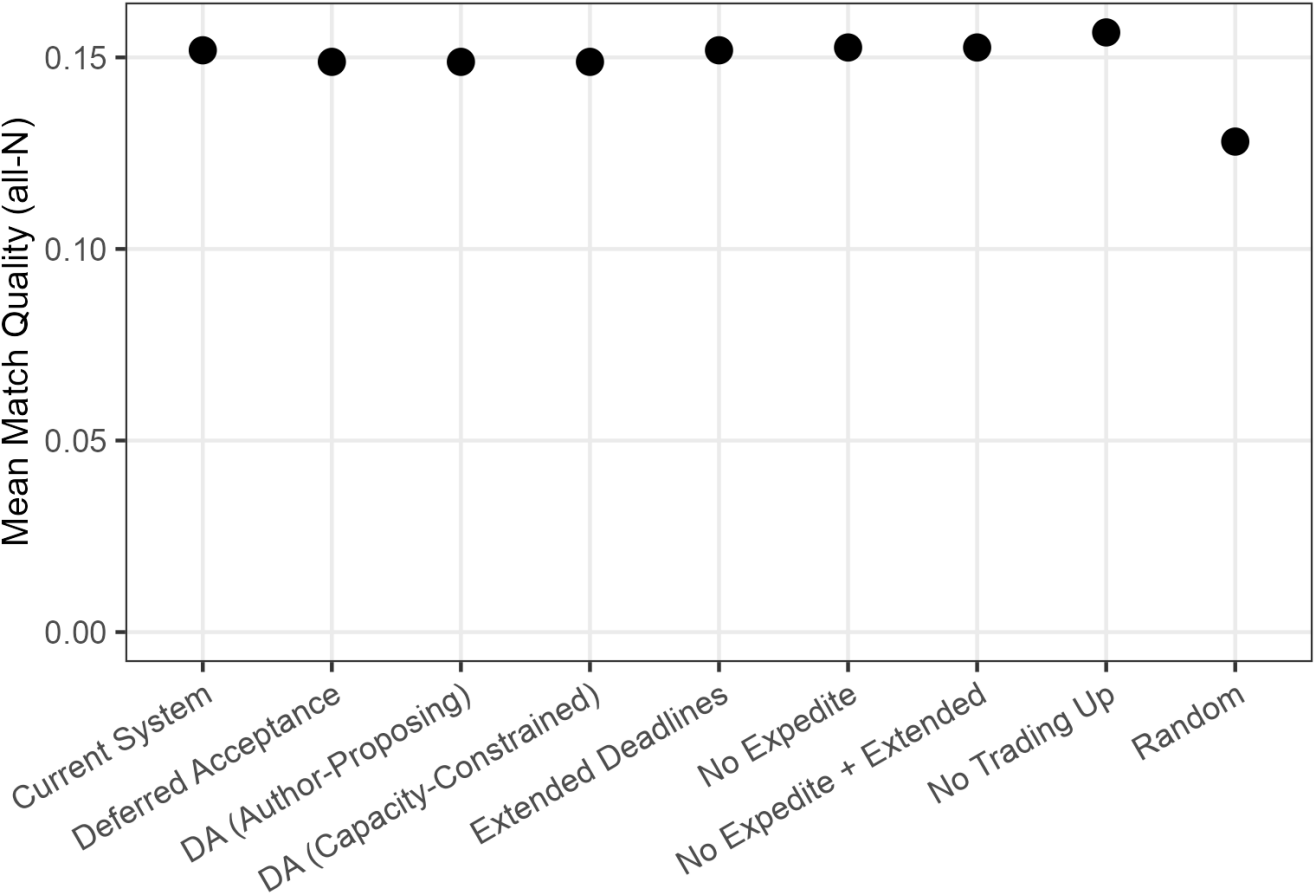
Aggregate match quality by mechanism. All-*N* welfare divides total match quality by all *N* = 800 articles, penalizing mechanisms that leave articles unplaced. The current system and its partial reforms cluster near each other, all modestly above the deferred acceptance (DA) benchmark. Random assignment provides a lower bound; no trading up achieves the highest all-*N* welfare by maximizing throughput at the cost of sorting.

However, the results also reveal a tension between sorting and *throughput*—the number of articles placed. The current system places approximately 50 more articles per simulation run than DA (488 versus 437). Because our overall match quality measure (Eq 5) counts unmatched articles as zero, this placement advantage can offset the sorting deficit. At baseline, the current system’s overall match quality is 0.152, compared to 0.149 for DA—a modest 2.0% advantage. This means that in moderately loose markets, the current system’s ability to place more articles can outweigh its substantially weaker sorting. The pattern reverses, however, when we consider only the articles that actually receive a placement: under the matched-only welfare metric $\bar{Q}(\mu )$, which averages quality of placement among matched articles and thus isolates sorting from throughput, DA consistently dominates (0.272 versus 0.249 at baseline, Table 2). The current system’s all-*N* advantage therefore reflects placements rather than placement quality: it depends on placing additional articles in lower-prestige journals where the per-article welfare contribution is small. Fig 3 provides a visual overview of the all-*N* welfare measure across all mechanisms.

The journal-proposing and author-proposing versions of DA produce identical outcomes across all 2,000 runs, meaning there is essentially only one stable matching in this market. This is expected given the preference structure: all authors rank journals by the same prestige ordering (within their submission ranges), and journals evaluate articles on a continuous signal that makes ties extremely rare. Together, these features collapse the lattice of stable matchings to a single point. This simplifies the analysis: we do not need to worry about which version of DA to use as the benchmark.

The counterfactual mechanisms provide insight into which institutional features drive the sorting–throughput tradeoff.

*Extended deadlines*. One might expect that giving authors more time to make decisions would reduce cascading and improve matching quality. However, replacing all journals’ heterogeneous deadlines with a uniform 14-day window produces results virtually identical to the current system (all-*N* welfare 0.1519 versus 0.1519, rank correlation 0.449 versus 0.449). Deadline pressure alone is not a meaningful source of misallocation.

*No expedite*. Removing queue-jumping from expedite requests modestly improves match throughput (490 matched versus 488) while slightly worsening rank correlation (0.434 versus 0.449). The all-*N* welfare rises to 0.1526, a 2.5% advantage over DA. Expedite removal helps by reducing cascading rejections, but the effect is modest.

*No trading up*. Requiring authors to accept their first offer dramatically increases throughput (543 matched) at a large cost to sorting (rank correlation 0.294). This mechanism achieves the highest all-*N* welfare (0.1565, + 5.2% over DA) precisely because it forces immediate clearance. It is analytically useful for isolating the placement-versus-sorting tradeoff but should not be interpreted as a desirable reform: its sorting quality is substantially worse than any other mechanism except random assignment.

*Random assignment*. As expected, random assignment produces near-zero rank correlation and very low match quality. It matches all 800 articles (the only mechanism to do so) because it imposes no selectivity. Its all-*N* welfare of 0.128 serves as a lower bound.

The decomposition (Eqs 6) breaks the total welfare gap between the current system and DA into contributions from each institutional feature. At baseline, the total gap *W*(DA) − *W*(Current) is −0.003, meaning the current system slightly outperforms DA on all-*N* welfare. Removing expedite queue-jumping improves welfare modestly ($\Delta_{\text{expedite}}>0$). Extending deadlines contributes less than 1%. The centralization step itself *lowers* all-*N* welfare ($\Delta_{\text{central}}<0$), because DA’s lower placement rate more than offsets its sorting gain at baseline. The vast majority of the difference thus comes from replacing the sequential, decentralized process with simultaneous evaluation, not from removing any specific institutional feature like expediting or deadline pressure. This pattern holds across all parameter variations reported below. Because the decomposition is path-dependent (performing the reforms in a different order would change the shares), these figures should be read as an accounting exercise rather than a causal attribution.

### Distributional effects

The overall averages mask important differences across the journal hierarchy. The decentralized market does not merely sort less well than centralized matching in the aggregate; it systematically compresses the quality distribution, pushing weaker articles into elite journals and stronger articles down into mid-tier journals.

**Fig 4:**
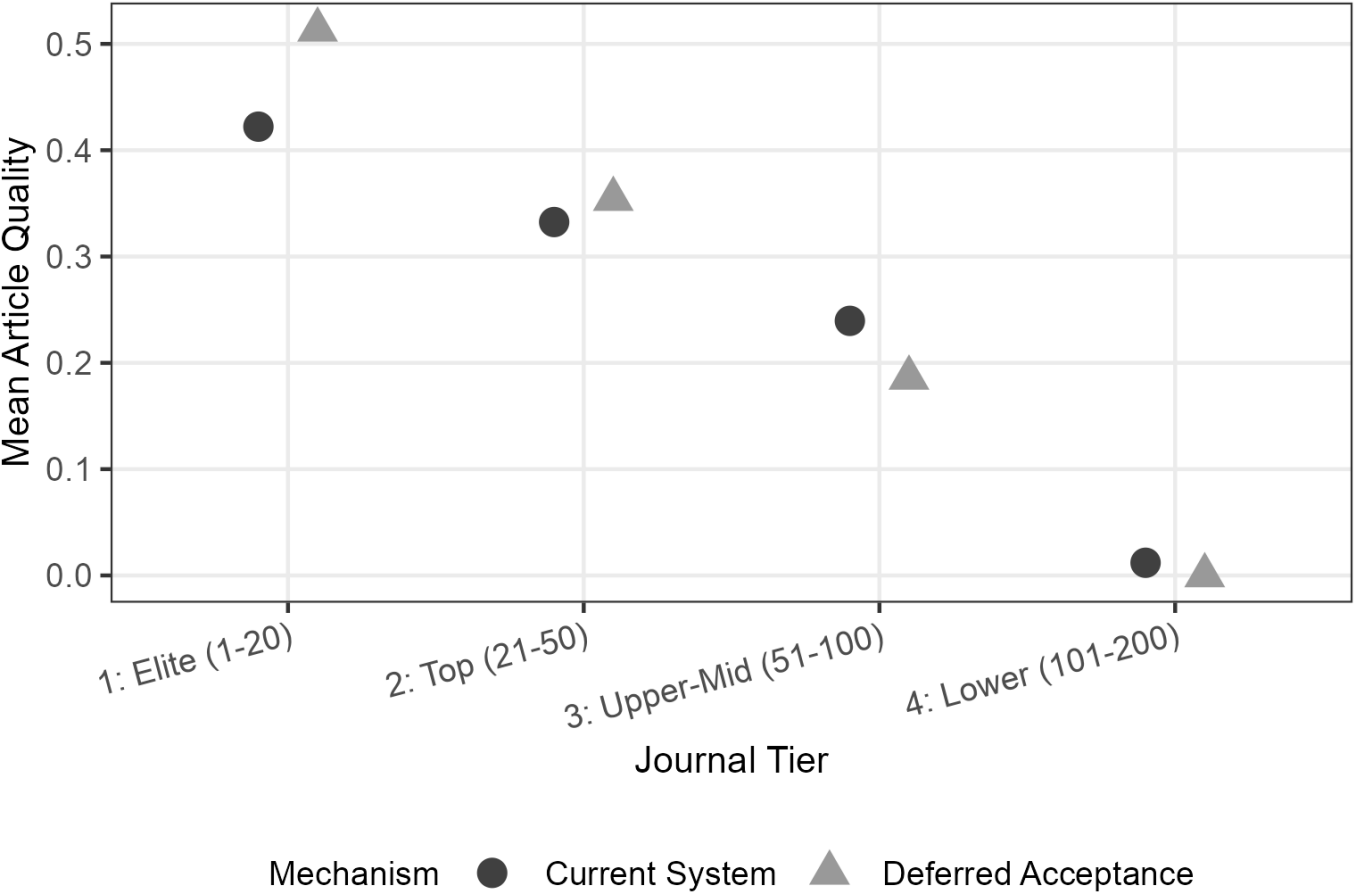
Mean article quality by journal tier under the current system and deferred acceptance. The current system compresses the quality distribution: elite journals receive weaker articles, while mid-tier journals receive stronger articles.

**Fig 5:**
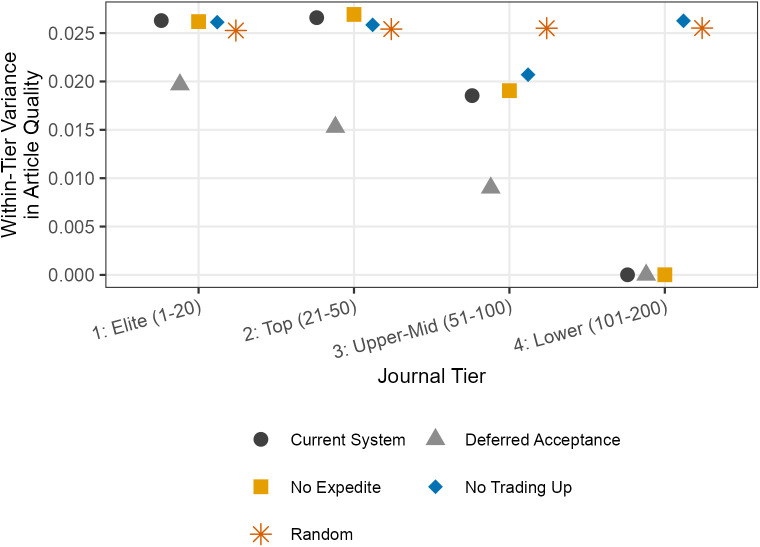
Within-tier variance in article quality under the current system and deferred acceptance (DA). Higher variance indicates less predictable placements within each tier.

Article quality at the top of the hierarchy suffers most. Elite journals (Tier 1, ranks 1–20) receive articles averaging 0.422 in quality under the current system versus 0.513 under DA—a large deficit (Fig 4). Tier 2 journals (ranks 21–50) show a similar pattern (0.332 versus 0.354), but Tier 3 journals (ranks 51–100) show the opposite: they receive stronger articles under the current system than under DA (0.240 versus 0.186). Good articles are displaced to lower-ranked journals while weaker articles fill elite slots. Within-tier variance is also consistently higher under the current system (Fig 5), indicating that placements are not only shifted on average but less predictable within each tier.

**Fig 6:**
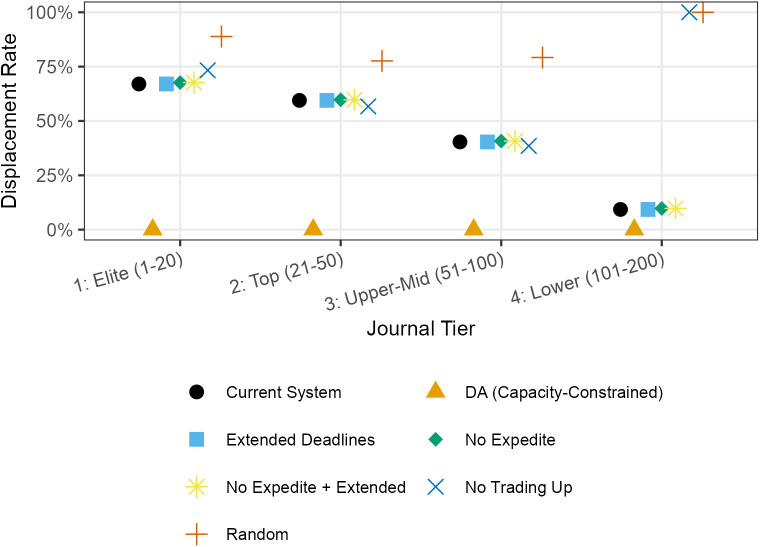
Displacement rates by tier. The fraction of placements under each mechanism that differ from the deferred acceptance (DA) benchmark. A displacement rate of 60% means that 60% of articles placed in that tier would land in a different tier under DA.

The mechanism behind this compression is the cascading dynamics of expediting and trading up. When a strong article is submitted, its author may receive an early offer from a mid-tier journal and hold it while requesting expedited review at elite journals. Meanwhile, weaker articles may fill slots at elite journals by arriving at the right time or benefiting from favorable expedite timing. For example, suppose all articles submit on day 1. Due to random queue ordering, a weaker article lands near the top of an elite journal’s queue and is reviewed on day 3, receiving an offer that fills a slot. A stronger article, lower in the elite journal’s queue, is meanwhile reviewed first by a mid-tier journal on day 5, which makes an offer. The author holds this mid-tier offer and requests expedited review at the elite journal, but by day 8, when the elite journal processes that request, no slots remain. The strong article ends up at the mid-tier journal; the weak article at the elite journal. These timing-driven misallocations cascade across the market, pushing the quality distribution downward. The displacement rates quantify just how much reshuffling occurs: approximately 67% of Tier 1 placements, 59.5% of Tier 2 placements, and 40% of Tier 3 placements under the current system differ from what centralized matching would produce (Fig 6). About two-thirds of articles placed in elite journals would not be there under centralized signal-based matching, a remarkably high rate showing that the decentralized market produces a fundamentally different allocation, not merely a slightly noisier version of the same one.

**Fig 7:**
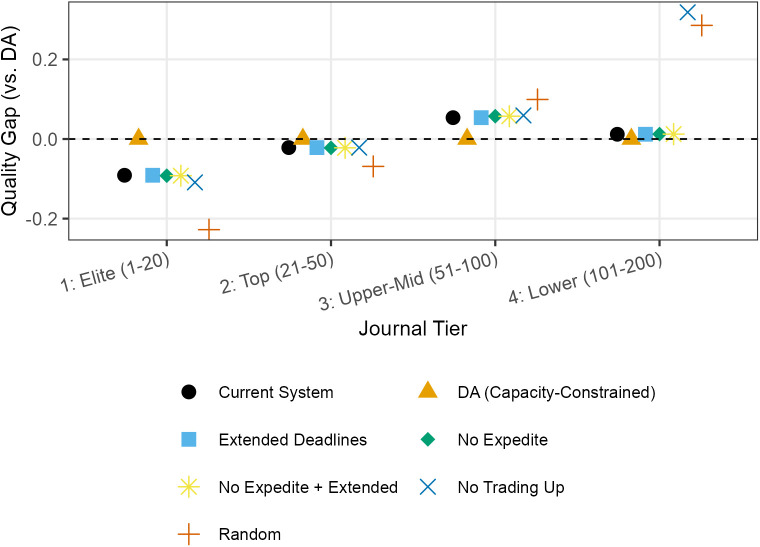
Quality gap versus deferred acceptance (DA) by journal tier and mechanism. Negative values indicate that the mechanism places weaker articles than DA in that tier; positive values indicate stronger articles. All decentralized mechanisms compress quality relative to DA, with the largest deficits at the top of the hierarchy.

This compression pattern is not a quirk of any single institutional feature. When we compare all decentralized mechanisms against DA (Fig 7), every one exhibits quality deficits at Tiers 1 and 2 and stronger-than-expected articles at Tiers 3 and 4. The “no trading up” mechanism produces the most extreme redistribution, and random assignment shows the largest Tier 1 deficit. Quality compression is thus a common consequence of decentralized clearing in the simulations.

**Fig 8:**
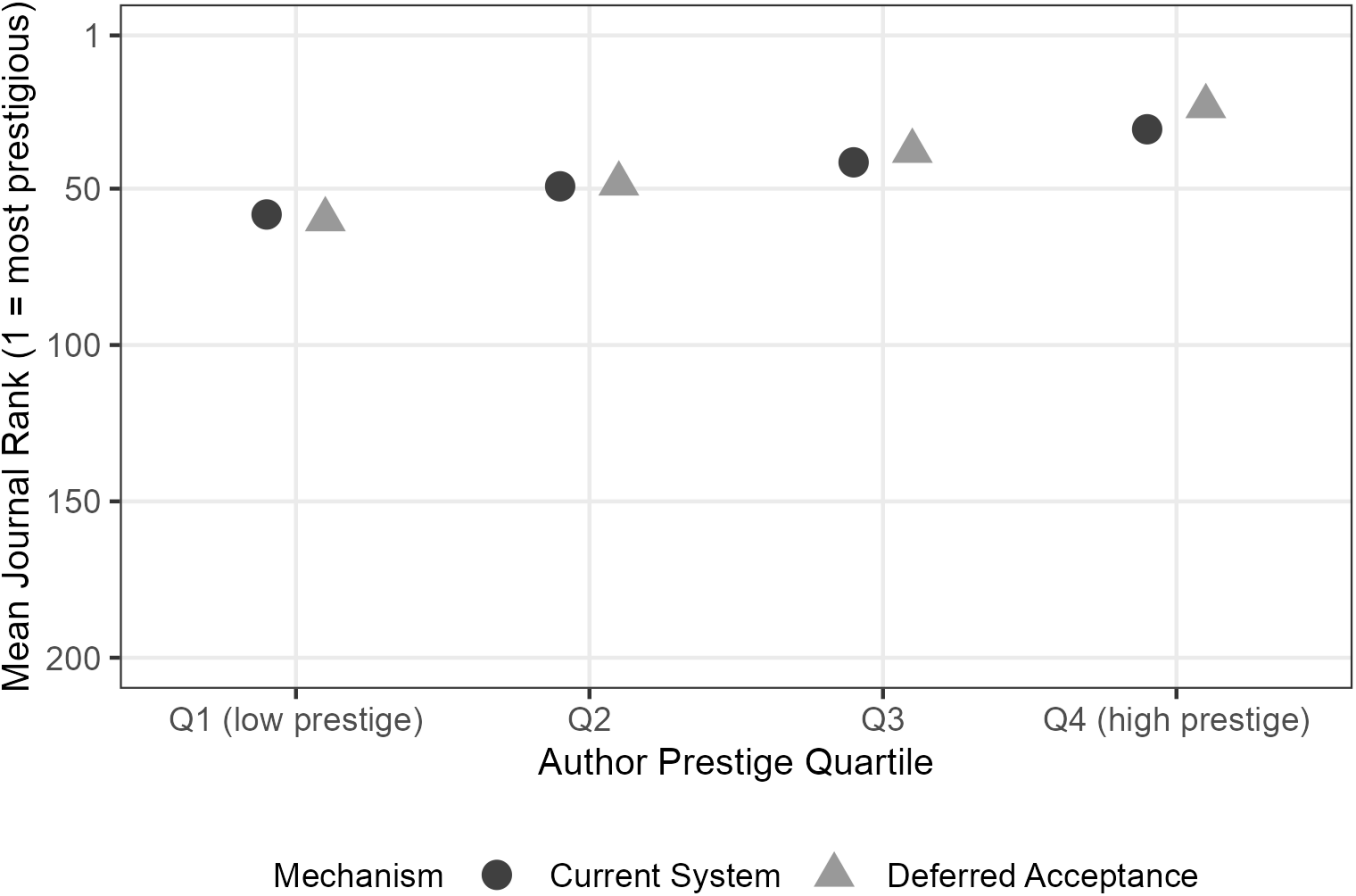
Mean placement rank by author prestige quartile under the current system and deferred acceptance (DA). Lower rank numbers indicate more prestigious placements. Authors are divided into four equal groups by prestige score.

The journal-side compression has a corresponding author-side consequence. The current system places high-prestige authors (Q4) in journals averaging around rank 31; DA places them around rank 24 (Fig 8). Low-prestige authors (Q1) show the opposite pattern: their average placement rank is around 58 under the current system versus around 60 under DA. The current system thus slightly reduces the placement advantage that high-prestige authors enjoy relative to DA. This is consistent with the quality compression pattern: when top journals receive weaker articles, some of the displaced strong articles come from high-prestige authors, who end up in less prestigious journals than they would under centralized matching.

**Fig 9:**
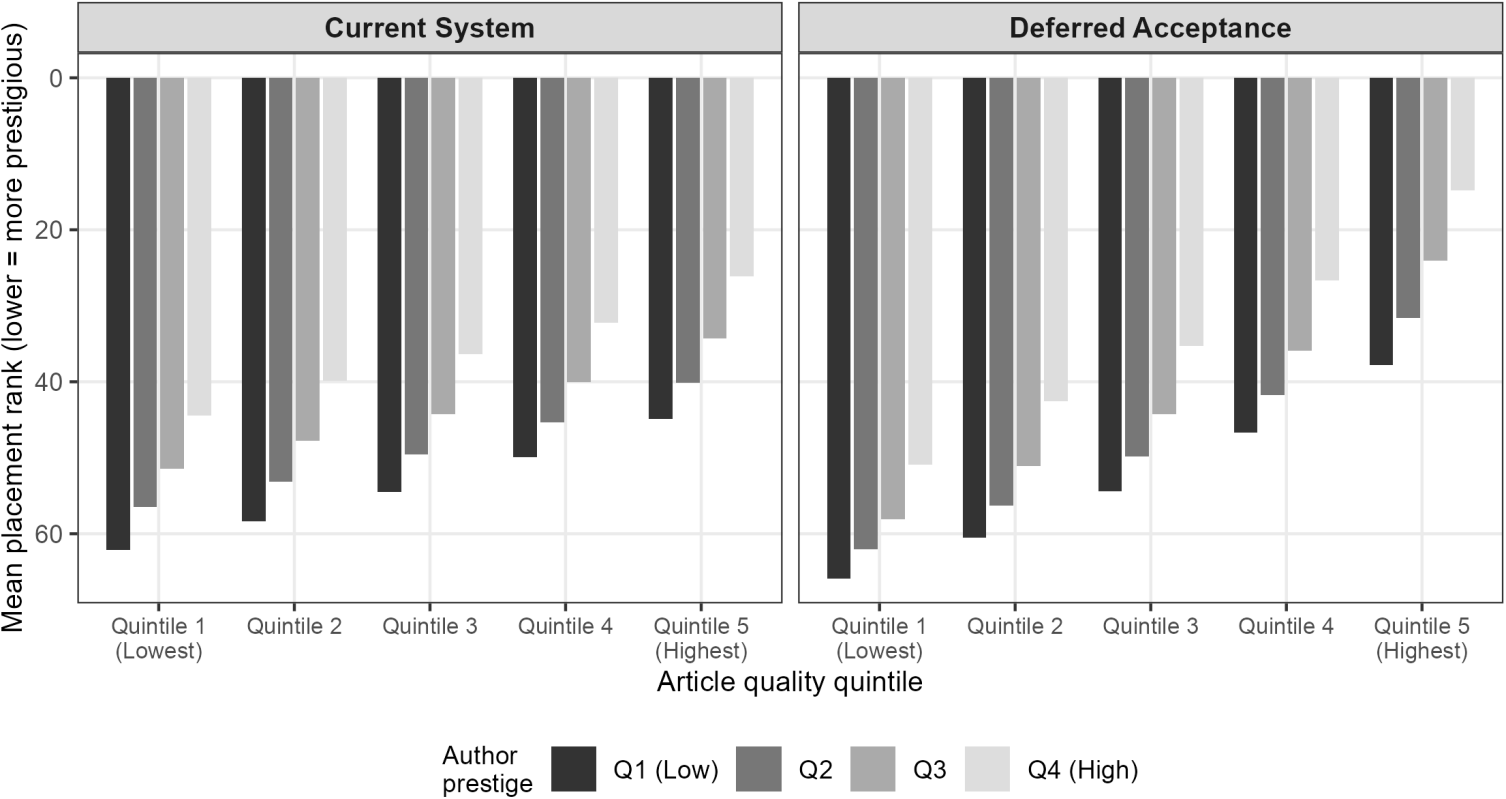
Conditional prestige advantage by quality quintile. Within each quality quintile, authors are divided into four prestige quartiles (Q1 = lowest, Q4 = highest). Bars show the mean journal rank of placement (lower = more prestigious). The steep prestige gradient within each quality bin shows that credential-based advantages persist even among articles of comparable quality, and are nearly identical under both the current system and deferred acceptance.

We next ask whether these prestige advantages persist after controlling for article quality. We bin articles into quality quintiles and, within each quintile, compare mean placement rank across prestige quartiles (Fig 9). Under both the current system and DA, high-prestige authors receive better placements than low-prestige authors *even among articles of comparable quality*. For example, among middle-quality articles (quintile 3), the placement gap between the lowest- and highest-prestige quartiles is approximately 18 ranks under the current system (around rank 55 for low-prestige vs. around rank 36 for high-prestige authors) and 19 ranks under DA (around 54 vs. around 35). The pattern is consistent across all five quality quintiles (Fig 9): within each quality bin, the prestige gradient is steep and nearly identical under both mechanisms. The conditional prestige advantage is comparable in magnitude across the two mechanisms, reflecting the fact that both rely on the same prestige-tainted signals (*w*_*j*_ < 1). Centralized matching improves sorting efficiency but does not address the inequity embedded in the signal structure itself. The finding confirms that the inequity documented in this paper is not merely an artifact of prestige–quality correlation: it persists within quality strata and would not be resolved by mechanism redesign alone.

We note that the within-quintile prestige advantage reflects two distinct channels: biased editorial signals (*w*_*j*_ < 1, which directly favor prestigious authors) and endogenous submission strategies (high-prestige authors submit to a narrower, higher-ranked set of journals). Decomposing these channels is an important direction for future work.

### Market tightness

How competitive the market is turns out to be the single most important factor in determining which system performs better. We vary the slot scale parameter λ from 0.25 (a very competitive market with only about 386 total slots for 800 articles) to 1.0 (the baseline, with approximately 1,545 slots). Table 3 summarizes the results.

**Table 3:** Market tightness sensitivity (expanded). The slot scale λ controls how many publication slots are available relative to submissions; lower values mean a more competitive market. “Gap” is the percentage difference in all-*N* welfare between the current system and deferred acceptance (DA). “Rank Corr.” is the Spearman correlation between article quality and journal prestige. “Tier 1 Qual. Gap” is the difference in mean article quality placed in elite journals (ranks 1–20) between DA and the current system. “Displ.” is the percentage of Tier 1 placements that differ from DA. Values are means across 500 simulation runs per setting.

**Panel A: Mechanism comparison across market tightness**								
		**All-*N* Welfare**			**Rank Corr.**		**Tier 1**	
** λ **	**Slots**	**Current**	**DA**	**Gap**	**Current**	**DA**	**Qual. Gap**	**Displ.**
0.25	386	0.0652	0.0753	–13.4%	0.516	0.821	0.111	79.6%
0.50	772	0.0990	0.1079	–8.2%	0.471	0.806	0.105	73.7%
0.75	1,159	0.1262	0.1302	–3.1%	0.450	0.796	0.098	70.1%
1.00	1,545	0.1521	0.1491	+2.0%	0.449	0.786	0.091	67.3%
**Panel B: Efficiency loss decomposition (% of total welfare gap)**								
Each row shows the share of the total gap *W*(DA) − *W*(Current) attributable to expedite removal, deadline extension, and centralization. At λ=1.0, the total gap is negative (the current system outperforms DA), so the centralization share is negative and large.								
** λ **	**Expedite**	**Deadline**	**Central.**					
0.25	32.4%	–7.0%	74.7%					
0.50	34.1%	–8.8%	74.7%					
0.75	36.7%	–3.1%	66.4%					
1.00	27.9%	0.1%	–128.0%					

As the market becomes more competitive, the current system’s performance deteriorates sharply relative to centralized matching. When publication slots are scarce, there is no room for error: a misallocated article that would have found a good home in a loose market now loses its best opportunity, and cascading rejections and overbooking churn amplify the damage. At λ=0.25, DA outperforms the current system by 13.4% on all-*N* welfare. At the baseline λ=1.0, the current system’s higher match rate offsets its sorting deficit. The rank correlation gap persists at every tightness level (Table 3): DA consistently achieves rank correlations near 0.8, while the current system remains near 0.45–0.52.

The decomposition is also informative across tightness levels (Table 3, Panel B). At λ=0.25, centralization accounts for the majority of the gap and expedite removal for a substantial secondary share. As the market loosens, the centralization component grows in magnitude beyond 100% (reaching –128% at λ=1.0), meaning DA’s lower match rate actively hurts on this metric; signed components are reported as percentages of the absolute total gap, so a negative share indicates a step that moves welfare in the opposite direction from the overall sign. The expedite component remains relatively stable across tightness levels.

The tier compression effect intensifies dramatically in tight markets. The Tier 1 quality gap and displacement rate both grow as the market tightens (Table 3), with the quality gap roughly 22% larger than baseline and displacement rates rising sharply at λ=0.25.

### Aspiration breadth

The aspiration scale parameter γ controls how selectively authors target journals—in other words, how willing they are to settle for a journal below their ideal. At low γ (0.2), authors aim narrowly at the most prestigious journals they think they can reach; at high γ (0.6), they spread their aspirations more broadly across the hierarchy. This parameter proves to be the second most important factor (after market tightness) in determining which system performs better. Table 4 summarizes the results.

**Table 4:** Aspiration breadth sensitivity. The aspiration scale γ controls how selective authors are: lower values mean authors hold out for more prestigious placements, higher values mean they settle more readily. “Gap” is the percentage difference in all-*N* welfare between the current system and deferred acceptance (DA). “Current Matched” reports the number of articles placed under the current system.

γ	Current All-*N*	DA All-*N*	Gap	Current Matched
0.2	0.1372	0.1484	–7.5%	441
0.3	0.1421	0.1492	–4.8%	452
0.4	0.1519	0.1489	+2.0%	488
0.5	0.1583	0.1494	+6.0%	511
0.6	0.1590	0.1483	+7.2%	522

The driver is the number of articles placed. When authors are highly selective (γ=0.2), the current system places only 441 articles versus DA’s 437—virtually the same—so DA’s superior sorting wins easily. When authors are less selective (γ=0.6), the current system places 522 versus 437, an 85-article gap that overwhelms its sorting deficit. Author selectivity thus determines the effective level of competition: narrow targets create congestion at the same journals, mimicking a tight market even when total slots are plentiful. This reinforces the market tightness finding—both parameters govern how competitive the market feels, and when the market is effectively competitive by either route, centralized matching dominates.

### The sorting–throughput frontier

Market tightness and aspiration breadth are the two parameters that most affect which system performs better. To map out their joint effect, we run the simulation across a fine two-dimensional grid: market tightness λ ranges from 0.20 to 1.10 (step 0.05) and aspiration breadth γ from 0.15 to 0.65 (step 0.025), for a total of 399 parameter combinations at 200 simulation runs each.

**Fig 10:**
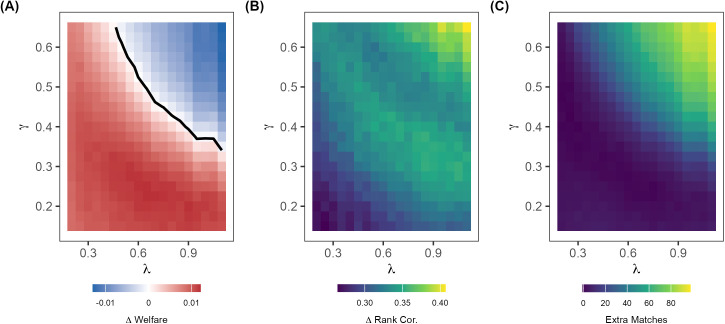
The sorting–throughput frontier across market conditions. Each panel varies market tightness (λ, horizontal axis) and author aspiration breadth (γ, vertical axis). Deferred acceptance is abbreviated DA. **(A)** DA welfare advantage (all-*N*): red indicates DA outperforms the current system, blue indicates the current system outperforms DA, and the white contour marks the breakeven boundary. **(B)** DA sorting advantage (Spearman rank correlation gap): DA sorts better everywhere, with the largest gap in loose markets with broad aspirations. **(C)** Extra placements by the current system relative to DA: the throughput advantage grows with market looseness and aspiration breadth.

Fig 10A presents the welfare heatmap. A diagonal contour separates red (DA better) and blue (current system better) regions, revealing a structural boundary. Below and to the left, where markets are tight and authors are selective, DA’s sorting advantage dominates. Above and to the right, where markets are loose and authors aim broadly, the current system’s throughput advantage dominates.

The companion panels (Fig 10B–C) show why the boundary falls where it does. DA sorts better everywhere, with the largest sorting advantage in loose markets with broad aspirations (upper right). But the current system also places the most extra articles in exactly that region. The welfare heatmap reflects the balance between these two forces: the boundary marks the combination of market conditions where the sorting gain from centralization exactly offsets the placement loss.

Taken together, the three panels reveal a *sorting–throughput frontier*: centralized matching improves sorting at the cost of fewer placements, while the current system places more articles at the cost of worse sorting. Which system produces more overall value depends on how competitive the market is and how selectively authors target journals.

### Robustness checks

We examine robustness to three additional factors that could influence the model’s conclusions: the strength of the relationship between author prestige and article quality (governed by the parameter ρ), the assumption about what happens when an offer deadline expires, and the addition of an explicit editorial-triage step. None alters the main findings (Table 5). We also use the ρ sensitivity to highlight an important property of the equity finding that is robust across the entire range of plausible prestige–quality correlations.

**Table 5:** Robustness checks. Panel A: Sensitivity to the prestige–quality mixing weight ρ. “Gap” is the percentage difference in all-*N* welfare between the current system and DA. “Matched” columns report the number of articles placed under the current system and DA respectively. Panel B: Sensitivity to deadline expiry behavior (accept versus lapse). Panel C: Editorial-triage robustness check. “Current + Triage” adds an explicit desk-rejection step in which each journal screens its initial queue at negligible cost (not consuming regular review capacity *c*_*j*_) and removes articles whose noisy signal falls below the 40th percentile of that journal’s queue. “Tier 1 Qual.” is the mean true quality of articles placed in Tier 1 (ranks 1–20). All other parameters held at baseline values. Values in Panels A and B are means across 200 simulation runs per setting; Panel C is means across 500 runs.

**Panel A: Prestige–quality mixing (**ρ)							
	**All-*N* Welfare**			**Rank Corr.**		**Matched**	
** ρ **	**Current**	**DA**	**Gap**	**Current**	**DA**	**Current**	**DA**
0.1	0.1490	0.1459	+2.1%	0.317	0.660	488	438
0.2	0.1504	0.1473	+2.1%	0.368	0.710	488	438
0.3	0.1506	0.1476	+2.0%	0.408	0.748	485	436
0.4	0.1531	0.1497	+2.3%	0.451	0.785	489	439
0.5	0.1525	0.1495	+2.0%	0.494	0.817	486	437
0.6	0.1539	0.1507	+2.1%	0.532	0.840	489	440
0.7	0.1542	0.1510	+2.1%	0.557	0.860	488	439
0.8	0.1549	0.1511	+2.5%	0.586	0.873	491	440
**Panel B: Deadline expiry behavior**							
	**All-*N* Welfare**			**Rank Corr.**		**Matched**	
**Behavior**	**Current**	**DA**	**Gap**	**Current**	**DA**	**Current**	**DA**
Accept	0.1518	0.1484	+2.3%	0.445	0.785	488	437
Lapse	0.1520	0.1492	+1.9%	0.446	0.787	488	438
**Panel C: Editorial triage**							
	**Mechanism**	**Matched-only**	**All-*N***	**Rank**	**Tier 1**	**Matched**	**Gap**
		**Welfare**	**Welfare**	**Corr.**	**Qual.**		**vs DA**
	Current System	0.250	0.1521	0.454	0.424	487	+2.1%
	Current + Triage	0.250	0.1526	0.421	0.427	488	+2.5%
	Deferred Acceptance	0.273	0.1489	0.788	0.515	437	0.0%

We vary the prestige–quality mixing weight ρ from 0.1 (where prestige is nearly uninformative about quality) to 0.8 (where prestige is a near-perfect indicator of quality). Results are stable across this entire range (Table 5, Panel A). The current system’s overall welfare advantage over centralized matching fluctuates between 2.0% and 2.5% with no systematic trend, and centralized matching consistently sorts much better than the current system regardless of how informative prestige is. The current system’s sorting problems therefore come from the mechanics of cascading offers and decentralized clearing, not because editors use prestige as a proxy. Even when prestige is an almost perfect indicator of quality, the current system still cannot match the sorting performance of centralized matching. In the model, the inefficiency is structural, not informational.

The robustness of the equity finding to ρ is worth highlighting separately. The conditional prestige advantage documented in Fig 9—in which high-prestige authors place markedly better than low-prestige authors *within* each true-quality quintile—remains pronounced even in the high-ρ regime (ρ=0.8), where prestige is a strong proxy for quality. The inequity we identify is therefore not an artifact of a weak correlation between prestige and merit. Even when most of an author’s prestige signal genuinely reflects article quality, prestige-tainted editorial evaluation (*w*_*j*_ < 1) continues to channel observably better placements to high-prestige authors at every quality level. The inequity is a robust feature of the evaluation process itself, and one that mechanism reform alone cannot resolve.

We also compare two assumptions about what happens when an offer deadline expires: either the author automatically accepts their current best offer (“accept” behavior), or the offer simply lapses, freeing the slot and returning the author to the submission pool (“lapse” behavior). Results are nearly identical under both assumptions (Table 5, Panel B), confirming that the model’s conclusions do not depend on this particular modeling choice.

Finally, we examine whether more aggressive editorial triage would narrow the gap between the current system and centralized matching. One might worry that the baseline model gives the decentralized system too little credit for editorial efficiency: in reality, student editors likely desk-reject a substantial fraction of submissions on a cursory glance, reserving careful review for the most promising manuscripts. The baseline model does incorporate a soft form of triage through quality-dependent review effort (Eq 3), with editors spending roughly 0.3 effort units rejecting weak articles and 1.0 on careful review of promising ones. However, weak articles still consume some capacity in the baseline and remain in the queue rather than being immediately desk-rejected. To test whether more decisive triage would change the results, we add an explicit desk-rejection step. Each journal screens its entire initial queue at negligible cost that does not draw from regular review capacity *c*_*j*_, and removes articles whose noisy signal falls below the 40th percentile of that journal’s queue. The regular review capacity *c*_*j*_ is then reserved for survivors; articles desk-rejected at one journal remain in other journals’ queues unchanged.

Results from 500 replications at baseline parameters (Table 5, Panel C) show that adding explicit triage produces essentially no change on aggregate measures: matched-only welfare 0.250 versus 0.250, all-*N* welfare 0.1526 versus 0.1521, articles matched 488 versus 487, and Tier 1 article quality 0.427 versus 0.424. The rank correlation between article quality and journal prestige is slightly *lower* under triage (0.421) than without it (0.454), because the noisy signal that drives editorial assessment also drives the desk-rejection decision. An article that draws an unlucky low signal at a given journal is removed from that queue entirely, and the trading-up process only partially recovers it across the market. The gap between the current decentralized system and centralized matching—DA rank correlation 0.788 and DA Tier 1 quality 0.515—persists essentially unchanged. The misallocation produced by the current decentralized system is therefore not an artifact of insufficient editorial triage; it is structural, and improving editorial efficiency at the screening stage does not by itself narrow the gap to centralized matching.

## Discussion and conclusions

The simulation yields a nuanced answer to the question of whether centralized matching would improve the law review market. We do not find that one system is universally better than the other. Instead, centralized matching does a far better job of placing the best articles in the best journals, while the current decentralized system places more articles overall. The sorting–throughput tradeoff arises because centralized matching requires that every placement be part of a *stable* assignment—one in which no unmatched journal–article pair would both prefer each other over their current options—and this stability constraint means that some articles the current system places through aggressive overbooking go unmatched under centralized matching. Which advantage matters more depends on how competitive the market is and how selectively authors target journals. The efficiency loss decomposition shows that the primary source of misallocation is the decentralized structure of the market itself, not any single institutional feature like short deadlines or expedite queue-jumping.

### Policy implications

Several policy-relevant conclusions follow from these findings. First, proposals to simply extend offer deadlines are unlikely to improve market efficiency. The simulation shows negligible effects from deadline extension across all parameter settings. The source of misallocation is not time pressure per se but the cascading dynamics that arise from simultaneous submission and trading up.

Second, the case for centralization is strongest when the market is competitive. If submission volumes continue to grow—as recent Scholastica data suggest [17]—the law review market would become effectively more competitive over time, even if journal capacity remains constant. Under those conditions, our model predicts the costs of decentralized clearing would increase. The simulation provides a quantitative basis for this claim: when we reduce available slots to one-quarter of their baseline level, the current system loses 13.4% of overall match quality relative to centralized matching. Arft-Guatelli and Baker [16] have independently proposed a detailed institutional design for a law review clearinghouse, drawing on the successful adoption of centralized matching in medical residencies and public school admissions; our simulation results provide the quantitative case for their proposal while also identifying an important limitation—the prestige bias embedded in editorial evaluation—that institutional design alone cannot address (see below).

Third, any reform proposal should be evaluated on both sorting and throughput dimensions. A mechanism that improves sorting at the cost of leaving many articles unmatched within the general journal segment may not be welfare-improving if those articles have no good outside options. Conversely, a mechanism that places many articles but sorts them poorly may degrade the informational value of journal prestige signals.

Fourth, mechanism redesign alone does not resolve the equity problem. Our conditional prestige-bias analysis shows that among articles of comparable true quality, high-prestige authors receive markedly better placements than low-prestige authors—and this gap is comparable in magnitude under both the current system and centralized matching. The source of this inequity is not the decentralized structure but the prestige-tainted editorial signals themselves (*w*_*j*_ < 1). In our model, any reform that improves how articles are *assigned* without changing how they are *evaluated* reproduces this bias. Addressing inequity therefore requires changes at the evaluation stage—such as blinded review, structured rubrics, or reduced reliance on author credentials—rather than at the assignment stage alone.

Fifth, the path from welfare improvement to actual adoption requires more than a favorable simulation result. Although our model shows that centralized matching improves aggregate sorting, the incentive structure facing individual journals is heterogeneous, and not all journals would benefit equally from reform. Elite journals (Tier 1 in our model) currently sit at the top of every cascade: they receive the strongest articles via expedite-driven trading up, face the lowest withdrawal risk, and enjoy the lion’s share of the most desired authors. Under centralization, they would still receive the highest-ranked articles by signal, but they would lose the cascade-generated information advantage and might receive fewer articles overall as throughput contracts toward the stable matching. Lower-ranked journals (Tiers 3 and 4) bear most of the cost of the current decentralized system: they expend editorial effort reviewing and offering on articles that are later withdrawn through trading up, and they must overbook aggressively to compensate. Centralization would reduce this churn and improve their effective hit rate, but it would also constrain them to articles their preference lists actually rank highly. Whether each tier supports reform thus depends on how it weighs these competing effects.

The same heterogeneity applies to exclusive-review tracks: elite journals lose the implicit quality signal carried by competing offers and gain only modest review-cost savings, whereas lower-ranked journals avoid the withdrawal cycle but may receive fewer high-quality submissions from authors who would otherwise have included them as part of a wide net. Historical experience suggests that voluntary, partial adoption is unlikely to produce a stable centralized market. The National Resident Matching Program, the canonical successful clearinghouse in the matching-market literature, achieved adoption only after a long period of unraveling chaos generated near-universal demand for coordination [9,12]. A clearinghouse for law reviews would face an analogous collective action problem: it requires broad participation to function, and unilateral adoption by a handful of journals would not by itself produce the welfare gains the model predicts. Reform proposals such as that of Arft-Guatelli and Baker [16] accordingly need to address not only mechanism design but also the institutional pathway by which a critical mass of journals could be brought into coordination—a challenge our model does not directly speak to but which the heterogeneous incentive structure makes apparent.

### Limitations and extensions

The model makes several simplifying assumptions, each of which also points toward a natural extension.

First, the model represents the general law review market of about 200 flagship journals, not the full universe of law journals. Scholastica currently lists roughly 540 specialty journals in addition to 195 general law reviews [17], and our *J* = 200 journals represent the general-interest segment. Specialty journals operate under partly different norms: topic fit matters more, submission volumes may differ, and the prestige hierarchy is less universally recognized. Our findings should not be extrapolated to that segment without further analysis. This restriction also affects how we interpret “unmatched” articles in our all-*N* welfare measure. Because the model represents only the flagship segment, articles we label unmatched are unplaced within that segment, not unpublished altogether—many would in practice find homes in specialty journals. If specialty placements provide an average prestige score comparable to the lowest-ranked flagship journals, where $g(r_{j})\approx 0$, then the zero-welfare penalty applied to unmatched articles in our all-*N* measure is a reasonable simplification. The welfare contribution of a specialty placement would already be approximately zero on our scale. To the extent that specialty journals provide meaningfully higher welfare, our all-*N* measure underestimates total welfare in absolute terms across all mechanisms, though the *relative* comparison between mechanisms is unaffected so long as the unmatched articles are similar across mechanisms.

Second, each author tracks only a single “best” offer and drops any previously held offer immediately when a better one arrives. In reality, authors may hold multiple live offers simultaneously, use lower offers as insurance or bargaining chips, and negotiate deadline extensions. The single-best-offer rule already captures the basic insurance and expedite dynamic, since authors can hold one offer while seeking better ones. What it omits is the additional option value and market-level congestion created when several offers remain live at once: authors can use multiple offers as parallel bargaining chips, tie up slots at several journals simultaneously, and manage different deadlines against each other. These dynamics could affect when cascades occur and whether they propagate primarily upward (toward more prestigious journals) or downward (as rejected authors seek alternatives at lower-ranked venues). The single-offer simplification therefore understates the strategic complexity of author decision-making and may compress the timeline of cascades. This makes the comparison between the current system and centralized matching *conservative*: a multi-offer model would likely exhibit even greater congestion and a larger DA advantage. Allowing authors to hold two or three live offers simultaneously would be a valuable extension.

Relatedly, the time-dependent acceptance threshold α(t) in Eq 4 is deterministic and identical across all authors, abstracting away heterogeneity in risk aversion. In reality, more risk-averse authors—for example, untenured scholars facing tighter promotion deadlines—may accept offers earlier than tenured authors, reducing the pool of articles engaged in strategic delay. A natural extension would let α(t) vary inversely with author prestige *p*_*i*_ to capture greater caution among lower-prestige and often more junior scholars, or otherwise introduce author-level heterogeneity in acceptance behavior; we leave this for future model refinement.

Third, when the simulation ends, journals that hold more articles than their slot count allows must shed the excess. The model resolves this with a stylized two-phase procedure (trim to capacity, then reallocate bumped articles in prestige order). This closure rule is a modeling device, not a description of a uniform real-world process; actual responses to overbooking are heterogeneous and may not involve structured reallocation at all. As noted in the Model section, this procedure is conservative: it benefits the decentralized mechanisms, so our comparison understates DA’s relative advantage.

Fourth, in the model, authors care only about journal prestige, and journals care only about a single score combining article quality and author prestige. Real preferences are more complex. Journals also care about topic fit, article length, issue balance, and sometimes substantive editorial priorities. This simplification is standard in the matching literature [11,12] and allows clean comparisons, but it means the model cannot capture inefficiencies that arise from mismatches along these other dimensions. If centralized matching improves topical fit more than prestige–quality fit, our results understate DA’s advantages; if the current system accidentally produces better topic matching through its decentralized dynamics, we overstate DA’s advantages.

Fifth, we measure efficiency by whether the best articles end up in the best journals. We chose this measure because it reflects the goal of a functional market: matching high-quality items with places where they are most valued. But it is a choice that reflects certain values, assuming that article quality and journal prestige are the only things that matter. In reality, other factors matter too, such as whether the journal is the right audience for the article’s topic, whether the article matches reader expectations, and what the author cares about beyond prestige. Our primary welfare measure also treats unmatched articles as contributing zero, which may bias results toward mechanisms that place more articles. We address this by reporting both overall and matched-only welfare throughout. Under matched-only welfare, centralized matching dominates everywhere, confirming that its sorting advantage is real and not an artifact of different placement rates.

Sixth, the DA mechanism uses the same noisy, prestige-tainted editorial assessments as the decentralized mechanisms, so it has no informational advantage. However, DA evaluates every article in each journal’s submission set simultaneously and ranks each queue by signal, whereas the decentralized mechanisms are bottlenecked by daily review capacity and process queues in submission order, modulated only by expedite priority. The DA comparison therefore reflects two distinct features of centralization—simultaneous clearing across the market *and* perfect ex-ante prioritization of each journal’s queue—and should be interpreted as an upper bound on what centralization alone could achieve without parallel reform of editorial workflow. Two robustness checks bound the importance of the prioritization channel. The capacity-constrained DA variant truncates each journal’s preference list to the top *K*_*j*_ articles it could feasibly review given its per-step capacity over *T* = 60 days; results are identical to unconstrained DA to the precision of our Monte Carlo estimates, because each journal’s cumulative review capacity over the full submission season already exceeds its submission pool size. More directly, the editorial-triage robustness check (Robustness checks section) gives the decentralized current system an explicit desk-rejection capability that removes the worst-signal articles from each journal’s queue—a realistic form of editorial prioritization, though weaker than DA’s full ex-ante ranking of survivors by signal. Even with this realistic triage in place, the gap to DA persists essentially unchanged, indicating that ordinary editorial triage is not sufficient to close the DA gap. A more complete test of DA’s perfect ex-ante prioritization would require a “decentralized full-information” variant in which each journal reviews all its submissions on Day 1 and ranks the surviving queue by signal before the trading-up process unfolds; we leave this for future work.

A separate caveat about the DA benchmark concerns author behavior rather than journal review. DA is constrained to authors’ original submission sets $\left[\ell_{i},h_{i}\right]$. A real clearinghouse that eliminated time pressure and trading-up incentives might also change author submission strategies—for instance, reducing the breadth of submissions—which would shift the sorting–throughput tradeoff in ways the current comparison does not capture.

Seventh, the submission density calibration validates the overall volume of submissions by journal rank but not the precise timing of editorial decisions. The aspiration scale parameter γ, a key moderator of which system performs better, is set by assumption rather than from direct empirical data on author behavior. Future work using submission-level data from platforms such as Scholastica could help calibrate both timing and aspiration parameters more precisely. The model also represents a single submission season with static journal capacity; it cannot capture how journal reputations change over repeated cycles or how past placement decisions influence future author behavior and editor expectations.

Several further extensions may merit pursuit. First, the editorial-triage robustness check we report uses a single triage threshold (40th percentile) and treats screening as negligible-cost; a fuller exploration over a grid of triage thresholds, and a variant in which the screening step itself consumes some review capacity, together with a Day-1 “decentralized full-information” counterfactual, would more completely isolate the role of editorial workflow within the decentralized system. Second, the current model treats expedite requests as pure queue-jumping; in reality, a competing offer also serves as an informational signal of article quality [3]. Modeling expedite as a Bayesian update to editorial assessments, and allowing authors to hold multiple offers simultaneously, would test whether the decentralized system performs better when operating at full realistic strength. Third, systematic sensitivity analysis over additional parameters (σ, *c*_*j*_, $\omega_{j}$, *w*_*j*_) beyond the aspiration scale and market tightness already explored would further map the robustness of our conclusions.

## Conclusions

Our model omits many real features of the law review market—topic fit, multi-round review, strategic deadline negotiation—but it captures the ones we believe matter most for placement outcomes: decentralized clearing, credential-based screening, compressed deadlines, and cascading expedite requests. We calibrated it to empirical data on submission volumes, editorial behavior, and institutional practices, and tested its conclusions across wide parameter ranges. The model predicts that the law review system produces substantial misallocation of articles to journals, and that credential-based advantages persist even among articles of comparable quality. The sorting–throughput tradeoff we identify may extend to other decentralized matching markets where participants submit to multiple options and decisions unfold sequentially, though confirming this would require studying those markets directly. As competition for journal space intensifies, the misallocation problem will grow worse; meanwhile, the equity problem persists regardless of market conditions because it is embedded in the signal structure itself. Fixing the misallocation requires changing how articles are assigned to journals; fixing the equity problem requires changing how they are evaluated. The two goals may interact: a system that reduces time pressure could also give editors more room to read carefully and rely less on credentials, narrowing the equity gap indirectly. Our model holds editorial signal weights fixed and therefore cannot capture this channel, but it identifies the target—if mechanism reform is to address inequity, it must ultimately change evaluation, not just assignment.
